# Kinetic characterization of annotated glycolytic enzymes present in cellulose-fermenting *Clostridium thermocellum* suggests different metabolic roles

**DOI:** 10.1186/s13068-023-02362-8

**Published:** 2023-07-12

**Authors:** Steve R. Daley, Patricia Mae Gallanosa, Richard Sparling

**Affiliations:** Department of Microbiology, University of Manitoba, 213 Buller Building, Winnipeg, MB R3T 2N2 Canada

**Keywords:** Metabolism, Bioprocessing, Glycolysis, Kinetics, Phylogeny, Evolution, PFK, *Clostridium thermocellum*, Enzymes

## Abstract

**Background:**

The efficient production of sustainable biofuels is important for the reduction of greenhouse gas emissions. *Clostridium thermocellum* ATCC 27405 is a candidate for ethanol production from lignocellulosic biomass using consolidated bioprocessing. Fermentation of cellulosic biomass goes through an atypical glycolytic pathway in this thermophilic bacterium, with various glycolytic enzymes capable of utilizing different phosphate donors, including GTP and inorganic pyrophosphate (PP_i_), in addition to or in place of the usual ATP. *C*. *thermocellum* contains three annotated phosphofructokinases (PFK) genes, the expression of which have all been detected through proteomics and transcriptomics. Pfp (Cthe_0347) was previously characterized as pyrophosphate dependent with fructose-6-phosphate (F6P) as its substrate.

**Results:**

We now demonstrate that this enzyme can also phosphorylate sedoheptulose-7-phosphate (an intermediate in the pentose phosphate pathway), with the *V*_max_ and *K*_m_ of F6P being approximately 15 folds higher and 43 folds lower, respectively, in comparison to sedoheptulose-7-phosphate. Purified PfkA shows preference for GTP as the phosphate donor as opposed to ATP with a 12.5-fold difference in *K*_m_ values while phosphorylating F6P. Allosteric regulation is a factor at play in PfkA activity, with F6P exhibiting positive cooperativity, and an apparent requirement for ammonium ions to attain maximal activity. Phosphoenolpyruvate and PP_i_ were the only inhibitors for PfkA determined from the study, which corroborates what is known about enzymes from this subfamily. The activation or inhibition by these ligands lends support to the argument that glycolysis is regulated by metabolites such as PP_i_ and NH_4_^+^ in the organism. PfkB, showed no activity with F6P, but had significant activity with fructose, while utilizing either ATP or GTP, making it a fructokinase. Rounding out the upper glycolysis pathway, the identity of the fructose-1,6-bisphosphate aldolase in the genome was verified and reported to have substantial activity with fructose-1,6-bisphosphate, in the presence of the divalent ion, Zn^2+^.

**Conclusion:**

These findings along with previous proteomic data suggest that Pfp, plays a role in both glycolysis and the pentose phosphate pathway, while PfkA and PfkB may phosphorylate sugars in glycolysis but is responsible for sugar metabolism elsewhere under conditions outside of growth on sufficient cellobiose.

**Supplementary Information:**

The online version contains supplementary material available at 10.1186/s13068-023-02362-8.

## Background

### Consolidated bioprocessing candidate

The recalcitrant nature of lignocellulose is an obstacle for current biofuel production strategies, relying heavily on yeast which is manifested in the form of higher operation cost associated with added cellulase cocktail and thermochemical pretreatment necessary for saccharification [1, 2]. Utilizing an organism that is capable of lignocellulose hydrolysis, followed by fermentation of the released mono- or oligomeric sugars using a consolidated bioprocess (CBP), would be a more cost-effective route [2, 3]. Among the known cellulolytic, ethanologenic bacteria, *Acetivibrio thermocellus* [4], but most commonly known as *Clostridium thermocellum* (and so named in the text) emerges as the front running CBP candidate, boasting the highest growth rate on crystalline cellulose [5, 6]; as well as the highest solubilization percentage of minimally pretreated mid-season switchgrass in comparison to other organisms[7], while fermenting the derived sugars to ethanol and acetate plus hydrogen (H_2_) [8].

### Deciphering glycolytic enzymes

*Clostridium* *thermocellum* is able to overcome the recalcitrance of potential feedstocks, but the ethanol yield is still below industry standards [9–12]. The low yield in ethanol can be attributed to three factors, the first being the branched fermentation pathway, which in addition to producing ethanol, also produces acetate, lactate, formate, H_2_ and carbon dioxide (CO_2_), pulling carbon and electrons away from ethanol [13, 14]. Advancement in system biology has led to improved metabolic models, which has exposed the two other factors including thermodynamic bottlenecks at key points in the glycolysis pathway and redox balancing centered around redox cofactors (NADH/NAD^+^, NADPH/NADP^+^, Fd(red)/Fd(ox) [15–17].

**Fig. 1:**
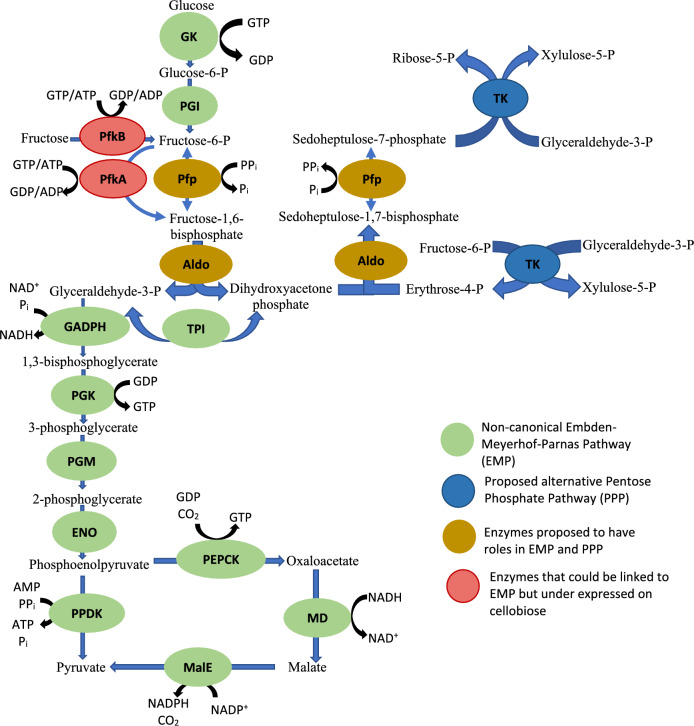
Proposed Embden–Meyerhof–Parnas pathway and alternative pentose phosphate pathway in *C. thermocellum.* Phosphoenolpyruvate can be converted to pyruvate either by the pyruvate phosphate dikinase (PPDK) or alternatively via the malate shunt pathway. GK glucokinase, PGI phosphate glucose isomerase, GAPDH glyceraldehyde-3-phosphate dehydrogenase, PGK phosphoglycerate kinase, PGM phosphoglycerate mutase, ENO enolase, PEPCK phosphoenolpyruvate carboxykinase, MDH malate dehydrogenase, MalE malic enzyme, OAADC oxaloacetate decarboxylase, TKT transketolase

The current study will pay attention to the kinetic characteristics of the enzymes involved in the upper glycolysis pathway with the pyrophosphate, ATP and GTP utilizing isozymes of phosphofructokinase (PFK) being the focal point, due to limited thermodynamic driving force at this point depending on the phosphate donor used [15, 16]; as well as the fructokinase and aldolase, responsible for production of the PFK substrate and subsequent cleavage of the product, respectively (Fig. 1).

PFKs are a diverse group of enzymes, all falling under one PFK superfamily, with different branches representing PFKs that are able to utilize different substrates, phosphate donors as well as being regulated by a number of effectors [18–20]. Within the superfamily, PFKs can be organized into three main phylogenetically distinct but related families. The Family A PFKs includes ATP-dependent PFK (EC 2.7.1.11) from higher eukaryotes and bacteria that are able to catalyze an irreversible catabolic reaction; as well as the PP_i_-dependent (PP_i_-PFK; EC 2.7.1.90) isoform belonging to bacteria, Archaea and in some cases plants, which catalyzes the reversible reaction, allowing the enzyme to function in both glycolysis and gluconeogenesis [18–21]. Members of this family are known to be regulated by multiple effectors, including ADP, fructose-2,6-phosphate and phosphoenolpyruvate [20, 22]. Family B PFKs exhibit phosphorylating activity with various sugar-based substrates including fructose (EC 2.7.1.4), adenosine (EC 2.7.1.20), fructose-1-phosphate (EC 2.7.1.56) and tagatose-6-phosphate (EC 2.7.1.144) [20, 21, 23, 24]. Finally, Family C PFKs are a rather small but distinctive family, which includes ADP-dependent PFKs belonging to thermophilic Archaea and some mesophilic methanogenic Archaea; organisms considered to represent the closest modern-day relatives of the last universal common ancestor [20, 21].

Fructose-1,6-bisphosphate aldolases (FBA) catalyze the reversible aldol condensation of dihydroxy-acetone phosphate and glyceraldehyde-3-phosphate to fructose-1,6-bisphosphate in glycolysis pathway [25]. FBA maybe segregated into two main classes depending on their molecular and catalytic properties [26]. Class I FBA, predominantly found in higher plants, animals, protozoa and green algae, catalyzes the reaction by forming a Schiff-base intermediate with the substrate via condensation of the ɛ-amine of a lysine residue in the active site [27–30]. Class II FBAs are characteristic of bacteria, fungi and blue-green algae [29, 30]. Class II FBAs utilizes a divalent cation (Zn^2+^, Fe^2+^ or Co^2+^) as an electrophile in their catalytic mechanism, which is further enhanced by addition of monovalent cations [26, 28, 30]. These requirements make class II FBA susceptible to inhibition by the addition of EDTA [26, 30]. Class II FBA can be further segregated into group A and group B. Group A is largely made up of FBAs that function in glycolysis and gluconeogenesis; whereas group B FBAs are more heterogenous with a more diverse range of metabolic roles and substrates to utilize including fructose-1,6-bisphosphate (FBP), tagatose-1,6-bisphosphate and sedoheptulose-1,7-bisphosphate [24, 30, 31].

*Clostridium* *thermocellum* has being shown to produce other valuable biomaterials such as isobutanol, 2,3-butanediol and 1-propanol during the fermentation of industrially relevant substrate concentrations, which requires metabolism through Embden–Meyerhof–Parnas (EMP) pathway [32]. Other industrially relevant organisms metabolize carbon through the (EMP) pathway that relies on different isoforms of PFK; nevertheless, remaining a key controller of glycolytic flux [15, 33, 34]. An ATP-PFK may appear more attractive as the isoform guarantees glycolysis in the forward direction required for subsequent biochemical production, but many organisms utilize PP_i_-PFK. Understanding the characteristics of PFKs from industrially relevant organisms and the associated effect on glycolysis and further biomaterial production from being ATP versus PP_i_-dependent would contribute to strain development.

Prior to the beginning of this study there were three genes putatively annotated as PFKs in the genome of the type strain *C*. *thermocellum* ATCC 27405/DSMZ 1237, including: an ATP-dependent PFK (Cthe_1261), a pyrophosphate (PP_i_)-dependent PFK (Cthe_0347) and another ATP-dependent PFK (Cthe_0389); as well as two FBAs (Cthe_0349 & Cthe_0319). All three PFKs were detected in the proteome, with the PP_i_-PFK being the prevalent isozyme translated in comparison to its supposed ATP counterparts, while only one of the FBA (Cthe_0349) was expressed [13]. Characterization of each enzyme has revealed its true identity and the role each would play in the atypical metabolism of *C. thermocellum*. Both furthering our understanding of the metabolic network and revealing how intertwined and dependent each pathway is to the other in the organism. These findings with respect to glycolysis to pyruvate are not only relevant for ethanol production, but also for the generation of any other end-product of potential interest.

## Results

### Purification of PFKs

**Fig. 2:**
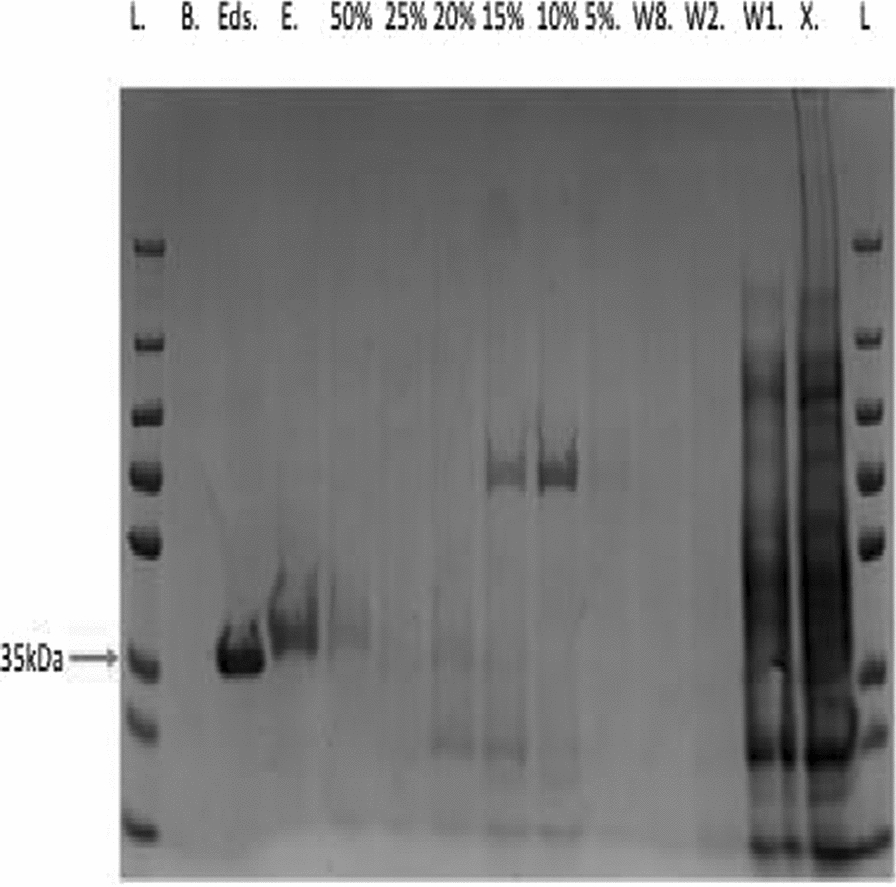
Purified recombinant protein, PfkA expressed in *E. coli* and recovered from the cell-free lysate after being passed through a HisTrap HP column charged with cobalt. Pure protein samples are seen in the lanes labeled Eds (eluted with 100% elution buffer & dialyzed) and E (100% elution buffer). Before elution of desired protein, the column was washed with 15 column volumes of binding buffer, followed by a 5% incremental increase of elution to binding buffer up to 50%. B.—water, X.— cell-free extract, W—binding buffer wash, L— prestained PAGE ruler plus protein ladder, #%- ration of elution to binding buffer in the wash

The monomeric molecular masses of the cloned and purified His-tagged PfkA and PfkB were approximately 40 kDa based off the SDS-PAGE, which is in close agreement with the molecular masses derived from the amino acid sequence, including the ~ 4-kDa linker and N-terminal histidine tag. Pfp in comparison was slightly larger with a monomeric molecular mass of approximately 50 kDA, which corresponds to the molecular mass derived from the amino acid sequence when the histidine tag is included. In regard to PfkA, a pure elution was obtained when the column was charged with cobalt rather than nickel as seen in the lanes labeled E. and Eds. (Fig. 2). Cobalt ions are more selective for histidine tags due to the requirement of adjacent histidine residues for binding in comparison to nickel ions; thus, the column was charged with cobalt to increase purity, rather than nickel which resulted in the co-purification of contaminants [35, 36]. Mass spectra analysis of all PFKs verified that the correct protein was eluted in each case.

### Kinetic characterization of PfkA

**Fig. 3:**
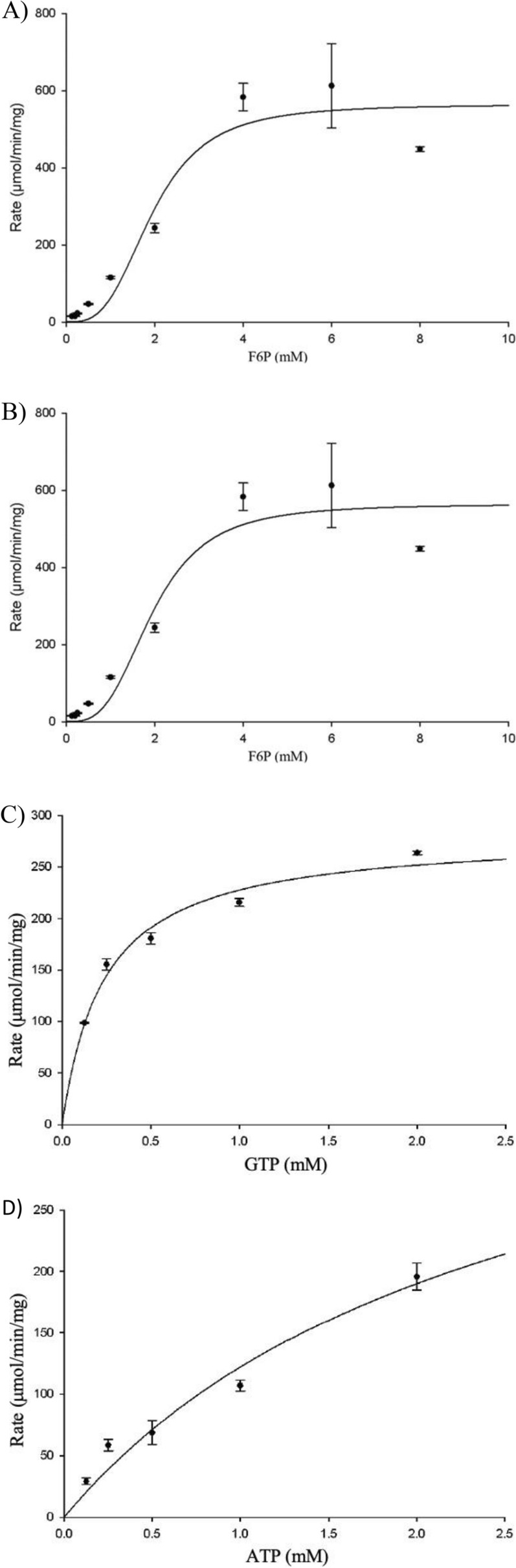
Both Hill and Michaelis–Menten kinetics of PfkA generated using SigmaPlot® 11.2. (**A**) Activity of PfkA versus varied F6P concentration fitted to Hill equation predicts a relatively high *K*_m_ of 1.9 mM for F6P, as well as *V*_max_, while GTP(2 mM) is used as a phosphate donor, in comparison to when (**B**) ATP(2 mM) is used fitted to Michaelis–Menten kinetics, producing approximately a third of the velocity, but a reduced *K*_m_ for F6P. (**C**) The *K*_m_ of GTP and (**D**) ATP fitted to Michaelis–Menten kinetics determined by varying the concentration of the phosphate donors while keeping the concentration of the substrate constant in the presence of NH_4_^+^. All assays were performed in triplicate

The recombinant PfkA displayed Michaelis–Menten kinetics with respect to both phosphate donors (GTP and ATP) and F6P when ATP was the phosphate donor, while kinetic assays for F6P (with constant [GTP]) fitted Hill equation in the presence of ammonium, which appears to be necessary for activity (Fig. 3). The kinetic parameters of the recombinant PfkA are summarized in Table 1 (see Additional file 6: Tables S4, S5). F6P appears to be the only sugar phosphate that PfkA utilizes as a substrate, as there was no product formation when the enzyme was challenged with either S7P (see Additional file 1) or fructose (data not shown). The enzyme appears to have a higher affinity for GTP as the phosphate donor in comparison to ATP which has a *K*_m_ value 12.5-fold higher than GTP (Fig. 3C, D). With a higher affinity for GTP, the enzyme produces the highest reaction rates when the substrate + phosphate tandem is GTP + F6P, which has been validated by the higher velocities observed when both phosphate donors are held at the same concentration (Fig. 3A, B). The highest *V*_max_ determined to be 563.9 µmol/min mg in assays involving GTP and F6P, was fitted to the Hills equation, giving a K_0.5_ value for F6P of 1.9 with a Hill coefficient (n) of 3 (Fig. 3A, B). There appears to be cooperativity with F6P when GTP is the phosphate donor, as opposed to ATP which fitted Michaelis–Menten kinetics.

**Table 1:** Kinetic characterization of recombinant Cthe_1261(PfkA)

$V_{\mathrm{max}}(\frac{\mathrm{\mu mol}}{\mathrm{mg min}})$	*K*_m_ (mM)	Phosphate donor^a^	Substrate^b^
430.5 ± 105.4	2.5 ± 0.96	**ATP**^**c**^	Fructose-6-phosphate
281.6 ± 8.9	0.2 ± 0.02	**GTP**^**c**^	Fructose-6-phosphate
169.1 ± 13.8	0.6 ± 0.1	ATP	**Fructose-6-phosphate**^**d**^
563.9 ± 42.4	1.9 ± 0.2	GTP	**Fructose-6-phosphate**^**d**^

^a^Phosphate donor used in the reactions

^b^Substrate used in the reactions

^c^The concentration of the phosphate donor was varied to determine the *K*_m_ of said phosphate donor, while the substrate concentration was held constant. Phosphate donors were used to initiate the reaction, indicated in bold

^d^The concentration of the substrate was varied to determine the *K*_m_, while the concentration of the phosphate donor was held constant at 2 mM. The assay for each concentration whether phosphate donor or substrate was done in triplicate. F6P was used to initiate the reaction, indicated in bold

There is a noticeable difference in the calculated *V*_max_ of the reactions containing both ATP + F6P (Table 1). One reaction assays for the *K*_m_ of ATP generating a *V*_max_ of 430.5 µmol/min mg, while the other reaction is measuring the affinity of F6P resulting in a *V*_max_ of 169. µmol/min mg (Table 1). The higher *V*_max_ recorded from the ATP affinity assay could be due to the fact Sigma-plot has to extrapolate pass the final ATP concentration (2 mM) used in the assay in order to determine the *K*_m_ of ATP(2.5 mM), accompanied by a higher standard deviation for the *V*_max_ (Fig. 3). While in the F6P affinity assays the lower *V*_max_ was determined at a constant ATP concentration (2 mM), therefore increasing the concentration of ATP may result in a comparable *V*_max_.

**Fig. 4:**
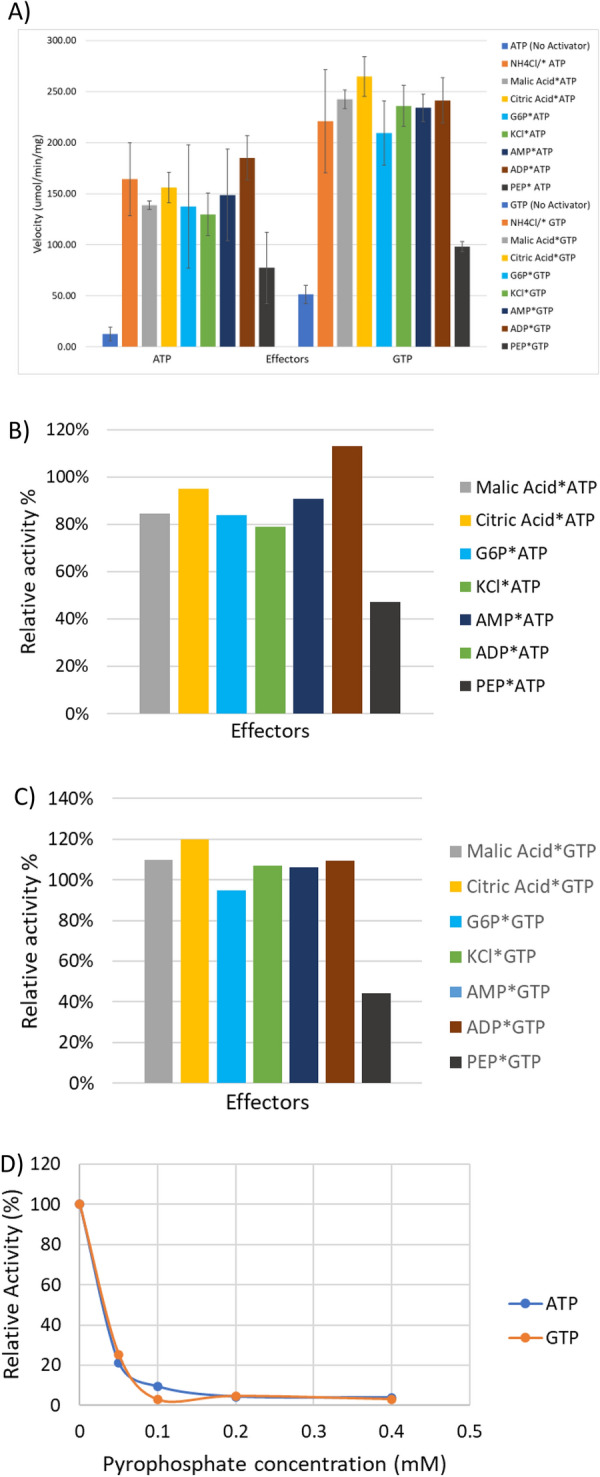
(**A**) Potential activating/inhibiting compounds added to the assays in addition to ammonium* (NH_4_^+^) which appears to be required by PfkA to produce substantial activity. The velocity of the enzyme in each reaction was recorded in the presence of 2 mM substrate, 2 mM phosphate donor and 2 mM effector. G6P-glucose-6-phosphate, PEP-phosphoenolpyruvate. (**B**) Relative activity of the enzyme with potential activating/ inhibiting compounds added to the assays in addition to ammonium* (NH_4_^+^) compared to assays with only NH_4_Cl added with ATP as the phosphate donor while (**C**) utilized GTP. (**D**) Relative activity of recombinant PfkA in the presence of various concentrations of PP_i_ for both phosphate donors. The concentration of Mg^2+^ in the assay was reduced 50% to 2.5 mM to avoid precipitation with PP_i._. All assays were performed in triplicate

Although GTP is the preferred phosphate donor, it appears to affect the affinity of the enzyme for F6P negatively in comparison to ATP. This phenomenon may be connected to how the enzyme is allosterically regulated within *C. thermocellum.* This may be linked to a change in conformation, that the enzyme transitions between when either GTP/ATP and their subsequent hydrolysis product (GDP/ADP) are present, thus affecting the cooperativity for F6P [22]. Previous studies have shown that ADP is capable of playing the role of an activator, thus lowering the *K*_m_ of F6P [22]. This may have been previously masked by the activation of NH_4_^+^. No activity was observed when ADP was the sole phosphate donor added to the reaction. Regardless of the phosphate donor, addition of PEP or PP_i_ caused a decrease in the activity of PfkA (Fig. 4) (see Additional file 6: Tables S6, S7), while addition of ADP did not significantly alter the activity. From the activity assays supplemented with PP_i_ (Fig. 4), it is apparent that concentrations of PP_i_ as low as 0.05 mM can cause a significant decrease in activity, with the relative activity decreasing to 21% and 25% for GTP and ATP, respectively, in comparison to assays without inhibitors. The *K*_m_ of PP_i_ for the highly expressed Pfp isoenzyme is 0.23 mM (Table 2), thus the likelihood of both these enzymes simultaneously phosphorylating F6P is very low. They are either expressed under different conditions or preferentially phosphorylate a sugar(phosphate) not tested in this study. All other effectors tested did not cause any meaningful change in activity.

**Table 2:** Kinetic characterization of recombinant Cthe_0347(Pfp)

Enzyme	Substrate/phosphate donor	$V_{\mathrm{max}}(\frac{\mathrm{\mu mol}}{\mathrm{mg min}})$	*K*_m_ (mM)
Cthe_0347	Fructose-6-P/**PP**_**i**_^a^	39.7	0.23^b^
	**Sedoheptulose-7-P**/PP_i_	6.294 ± 3.723	3.236^e^
	**Fructose-6-P**^**c**^**/** PP_i_	100.222 ± 11.6	0.075^d^

^a^This assay was done at 50 °C by [37] in comparison to ^c^ done in this study. ^b^*K*_m_ of pyrophosphate. ^d^*K*_m_ of fructose-6-P, ^e^*K*_m_ of sedoheptulose-7-phosphate while PPi-pyrophosphate (PP_i_) concentration was held constant

**Fig. 5:**
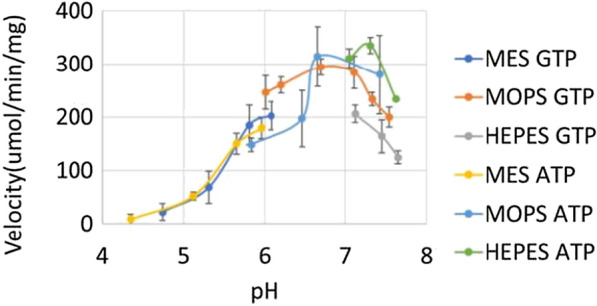
The pH range over which PfkA showed activity with both phosphate donors and F6P as the substrate. In order to reduce confounding effects only sulfonic acid buffering agents with varying pK_a_ were used. The velocity of the enzyme in each reaction was recorded in the presence of 2 mM of both the substrate and respective phosphate donor. Each assay condition was performed in triplicate

The optimal pH of PfkA appears to be 7.31 when ATP is utilized in comparison to 6.7 when GTP is used as the phosphate donor. Regardless of the phosphate donor, the enzyme does show substantial activity in the pH range of 5.12–7.65 (Fig. 5).

### Kinetic characterization of Pfp

**Fig. 6:**
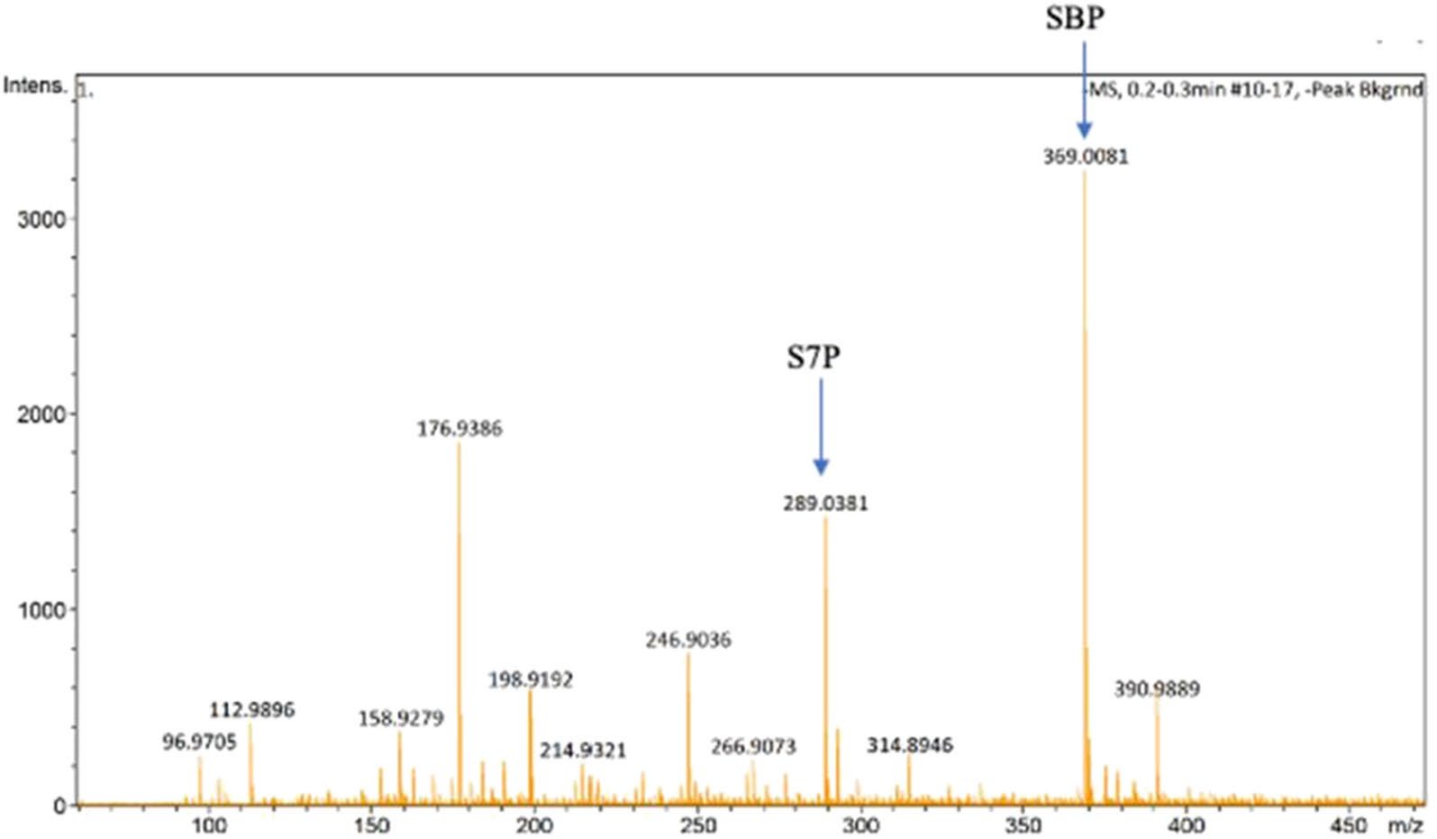
Mass spectrometry results supporting the phosphorylation of sedoheptulose-7-phosphate (S7P, MW = 290.162 g/mol) to sedoheptulose-1,7-bisphosphate (SBP,MW = 370.14 g/mol) by Pfp, which has previously shown activity with fructose-6-phosphate and pyrophosphate as the phosphate donor

The recombinant Pfp displayed Michaelis–Menten kinetics for both F6P and S7P when PP_i_ was utilized as the phosphate donor. A summary of the kinetic parameters is displayed in Table 2. It appears that Pfp is both more active and has a higher affinity for F6P in comparison to S7P. The Michaelis–Menten plot predicts a *K*_m_ for S7P of 3.24 mM, however, the highest concentration of S7P that could be used in this set of assays was 2.12 mM (see Additional file 2). Mass spectrometry analysis of the reaction products confirmed that S7P was being phosphorylated to SBP by Pfp in the presence of PP_i_ (Fig. 6). Activity assays were done with F6P alone as well as equimolar amounts of F6P and S7P, which showed a decrease in activity when both substrates were present in the assay as opposed to solely F6P (see Additional file 2: Table S3). This assay strictly monitored the production of FBP from phosphorylation of F6P, thus it is assumed that a decrease in activity was due to competition between F6P and S7P for the active site of the enzyme. Further kinetic characterization was done [37], but activity with phosphate as observed with the Pfp of *M. capsulatus, R. rubrum, X. campestris pv., A. methanolica, E. histolytica* (Table 4), was not investigated.

### Comparative structure–function relationship from primary sequence alignment of Family A PFKs

#### Substrate binding

**Fig. 7:**
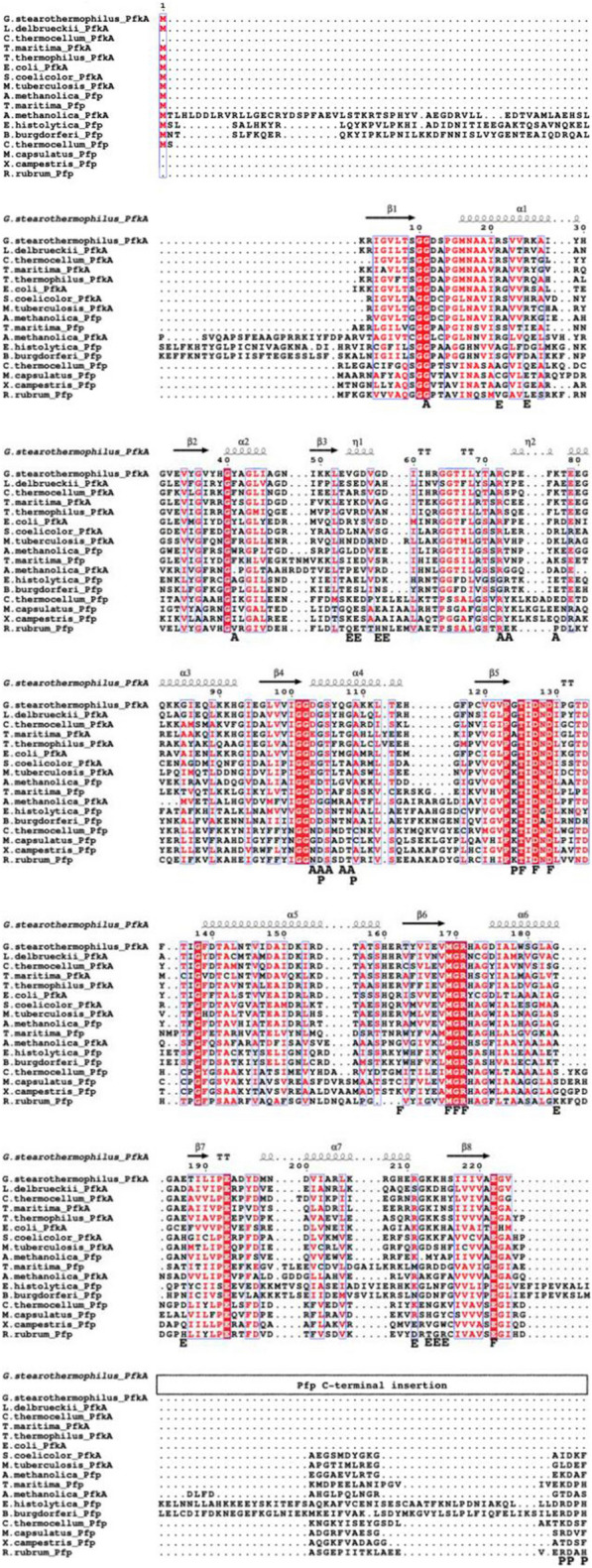
Amino acid alignments of Family A PFKs including both ATP-PFKs and PP_i_-PFKs, that have been purified and characterized to phosphorylate sugar phosphates with various phosphate donors. Protein crystal structures have been solved for *E. coli* PfkA, *G. stearothermophilus* PfkA and *B. burgdorferi* Pfp which aided in identifying residues implicated in binding F6P (**F**), ATP (**A**), PP_i_ (**P**) and effectors (**E**). The secondary structures displayed above the alignment was based off the secondary structures determined from the crystal structure of *G. stearothermophilus* PfkA, thus similar folds should be present in the highly conserved region of the other PFKs. The percent similarity between C. *thermocellum* PfkA and each of the other PFKs were placed at the end of the alignment, while percent similarity compared to *C. thermocellum* Pfp was placed in parentheses. Segments highlighted in red, identical residues, while letters in red represent similar residues. The sequences were aligned with MAFFT and the figure prepared with ESPript. Note that the PfkA sequences were truncated to focus on the N-terminal which contains the binding domains, whereas majority of the Pfp sequence was used during alignment. The amino acid numbering in the alignment corresponds to *E. coli* PfkA

As previously mentioned, Family A PFKs include both PfkA and Pfp. An alignment between characterized PfkA and Pfp proteins (Table 4) was done to deduce the residues necessary for binding phosphate donors, sugar phosphates and effectors based off the protein structure solved for *E. coli* PfkA and *B. burgdorferi* Pfp [38, 39] (Fig. 7). Residues highlighted in red represent identical residues, highly conserved for both families of PFKs, which are residues involved in the binding of F6P, a substrate that both PFKs are able to phosphorylate. All 11 residues required for binding F6P in *E. coli* PfkA, were also present in *C. thermocellum* PfkA, on the other hand it appears that *C. thermocellum* Pfp shares 9 out of 11 of those residues. The basic Arg162 in *E. coli* PfkA has been switched to methionine (neutral nonpolar R group) in *C. thermocellum* Pfp and tyrosine (neutral polar R group) in *B. burgdorferi,* while the basic His249 in *E. coli* PfkA was replaced with a tyrosine in *B. burgdorferi* and leucine (neutral nonpolar R group) in *C. thermocellum* Pfp. Similar replacements are seen in the Pfps of *E*. *histolytica* and *M. capsulatus* with a substitution of the basic residue, R162Y and R162C, respectively*,* as well as replacing H249Y. Both Pfps can phosphorylate S7P [40, 41], as seen with the Pfp of *C*. *thermocellum* in this study. The residue changes mentioned above are implicated in the binding of the 6-phosphate of F6P in *E. coli* PfkA [38]. The replacement of these residues in the Pfps in comparison to *E. coli* PfkA could provide some flexibility in regard to substrate binding but cannot be used as a conclusive reason as to why *C. thermocellum* Pfp is able to utilize both F6P and S7P.

The highly conserved residues encountered in all members of the PfkA family seem to be necessary in binding the sugar phosphate in the right orientation, with respect to magnesium and the phosphate donor [38, 39]. The general base, Asp127, removes the proton from the 1-OH group on the sugar phosphate, thus priming the substrate for a nucleophilic attack on the terminal phosphate of ATP or PP_i_ [38, 39]. This residue is required to enhance nucleophilicity regardless of the substrate. Cofactors such as Mg^2+^ and in the case of *C. thermocellum* PfkA, NH_4_^+^ are required to interact with ASP129. This residue is involved in binding said cofactors, which assist in the polarization of the phosphoanhydride bond to facilitate bond breakage [38, 42].

Tagatose-6-phosphate, another potential substrate of PfkA, supported by kinetic data of the PfkA belonging to *M. tuberculosis* (Table 4) [43], does raise the question if T6P is also a substrate of *C. thermocellum* PfkA and if there are conditions under which accumulation of T6P will trigger the expression of said PfkA. Such an assumption would be dependent on *C. thermocellum* having all the other required enzymes to produce T6P, which goes beyond the scope of this paper. The alignment of PfkA between *C. thermocellum* and *M. tuberculosis* (Fig. 7) show no discrepancy in regard to residues that bind the substrate. A protein structure of *M. tuberculosis* PfkA has being predicted with AlphaFold [44], but residues responsible for binding substrates were not identified. Although the alignment (Fig. 7) shows identical residues required to bind F6P for both organisms, the phosphorylation of T6P by *C. thermocellum* PfkA was not tested.

#### PP_i_ versus NTP binding in PFKs

There are two secondary structures and individual residues that influences PFK ability to bind either PP_i_ or ATP. PP_i_-PFKs have a large insertion on the C-terminal (between residues 220–230) relative to the PfkA of* E*. *coli* and other ATP-dependent PFKs. Part of this insertion forms a β hairpin which includes residues conserved among Pfps [39]. Out of the 6 conserved residues identified in *B. burgdorferi* that aid with binding PP_i_, *C. thermocellum* Pfp contains 2 identical and 1 similar residue (Fig. 7). These conserved residues are identified in the insertion region of the alignment with the letter ‘P’ below the alignment.

ATP-dependent PFKs lack this C-terminal extension; instead, there is a short loop starting at position 72 as seen in *E*. *coli* [38] and identified in the other ATP-dependent PfkAs in the alignment. There appears to be three residues that are required for binding ATP based on the *E*. *coli* PfkA structure, including Arg72, Phe73 and Arg77. *C. thermocellum* PfkA has an identical residue at position 72, a similar replacement at position 77 (R77K) and serine at position 73 instead of phenylalanine.

Other than the aforementioned structures, there are individual residues that predict if the enzyme is able to bind nucleotide triphosphate or PP_i_ based on steric hindrance [39]. The glycine residue at position 108 of *E*. *coli,* that is conserved among multiple PfkAs in the alignment, allows the adenine ribose moiety of ATP to fit into the active site, but the equivalent asparagine in *B. burgdorferi* or threonine in *C. thermocellum* Pfp is larger than glycine. The larger residues sterically prevent the adenine ribose moiety of ATP from binding at this site in Pfps, but leave enough room for the smaller PP_i_ molecule. A similar scenario takes place at position 104, as glycine is observed for all ATP-PFKs, as opposed to aspartate seen in PP_i_-PFKs. The residue at this position would come in close proximity to the α-phosphate binding pocket; therefore, only glycine would allow the binding of nucleotide triphosphate, in comparison to the negative aspartate which both sterically and electrostatically prevent the binding of the negative phosphate group [39]. This explains why *C. thermocellum* PfkA can utilize both ATP and GTP (the purine bases). However, the possibility to utilize UTP, CTP and TTP as seen in *Thermotoga maritima* PfkA [45] cannot be excluded at this time.

#### Effector binding

PP_i_-PFKs are generally not affected by common allosteric effectors, but ATP-PFKs tend to be regulated [20]. Multiple residues have been implicated in forming the allosteric effector site, deduced from the structure *E*. *coli* PfkA [38]. Activity assays conducted with *C. thermocellum* PfkA revealed that this enzyme, in agreement with some of its distance relatives, is noticeably inhibited by both PEP and PP_i_ (Fig. 4). Among the multiple residues that form the allosteric site, it appears that the glutamic acid residue at position 187 plays an important role in the inhibition of PFK by PEP and in activation by ADP or GDP [46]. The PfkA sequence alignment (Fig. 7) shows the conservation of this residue in most ATP-PFKs, but there are cases in which this residue is replaced by aspartic acid. The replacement E187D in mutant *E*. *coli* PfkA causes the enzyme to become insensitive to PEP, but still show stimulation with GDP comparable to wildtype [46]. However, replacement of Glu187 with a neutral residue such as leucine completely abolishes activation of GDP; due to the requirement for a negative charge at this position, which binds the magnesium ion of the (GDP)ADP-Mg^2+^ at the effector site [38, 46]. The presence of these residues does not guarantee activation or inhibition by ADP (GDP) or PEP, respectively, as verified by the fact that *C. thermocellum* PfkA showed a modest increase in activity with ADP, while its PP_i_-dependent counterpart that has an aspartic acid residue at position 187 (Fig. 7) is negligibly affected by the addition of 1 mM of ADP and PEP [37].

#### Kinetic characterization of PfkB

The recombinant PfkB displayed Michaelis–Menten kinetics with respect to fructose and both phosphate donors (GTP & ATP). A summary of the kinetic parameters is displayed in Table 3 (see Additional file 6: Tables S8, S9).

**Table 3:** Kinetic characterization of recombinant Cthe_0389(PfkB)

$V_{\mathrm{max}}(\frac{\mathrm{\mu mol}}{\mathrm{mg min}})$	*K*_m_ (mM)	Phosphate donor^a^	Substrate^b^
110.5 ± 12.1	0.4 ± 0.1	**ATP**^**c**^	Fructose
123.9 ± 14.3	0.2 ± 0.08	**GTP**^**c**^	Fructose
278.4 ± 57.5	1.8 ± 0.6	ATP	**Fructose**^**d**^
217.5 ± 20.4	0.7 ± 0.2	GTP	**Fructose**^**d**^

^a^Phosphate donor used in the reactions

^b^Substrate used in the reactions

^c^The concentration of the phosphate donor was varied to determine the *K*_m_ of said phosphate donor, while the concentration of the substrate was held constant at 2 mM. Phosphate donors were used to initiate the reaction, indicated in bold

^d^The concentration of the substrate was varied to determine the *K*_m_, while the concentration of the phosphate donor was held constant at 2 mM. The assay for each concentration whether phosphate donor or substrate was done in triplicate. F6P was used to initiate the reaction, indicated in bold

The enzyme can utilize either ATP or GTP during the phosphorylation of fructose, while no activity was detected with either S7H or F6P (see Additional file 3). GTP appears to be the preferred phosphate donor, with a *K*_m_ of 0.19 mM, in comparison to ATP with a *K*_m_ two-fold higher. Phosphorylation with ATP as the donor seems to decrease the enzyme affinity for fructose in comparison to when GTP is used as the sole phosphate donor, validated by a 2.5-fold difference in *K*_m_ values for fructose. The reason for the difference in fructose *K*_m_ depending on the phosphate donor is unclear. One possibility may be that there is a change in enzyme conformation depending on the phosphate donor used, affecting the interaction with fructose. Regardless of the phosphate donor, it is apparent that the enzyme is capable of velocities above 100 μmol/mg/min. This value becomes important when the *V*_max_ of the encoded Pfp (Table 2) is taken into account. If F6P was also being produced via PfkB under cellobiose/avicel grown conditions, there would likely be an even greater accumulation of the sugar phosphate, than what already occurs [16]; due to the higher velocity of the PfkB versus the Pfp. However, this is not the case as PfkB is not expressed significantly under cellobiose/avicel grown conditions [13]; therefore, F6P flux should either come through the annotated glucose-6-phosphate isomerase or from reactions in the pentose phosphate pathway. If PfkB were to be expressed as seen in *C*. *thermocellum* DSM 1313 [47], would this cause the encoded PfkA with a velocity in line with that of PfkB to be expressed and relieve the accumulation of F6P?

**Fig. 8:**
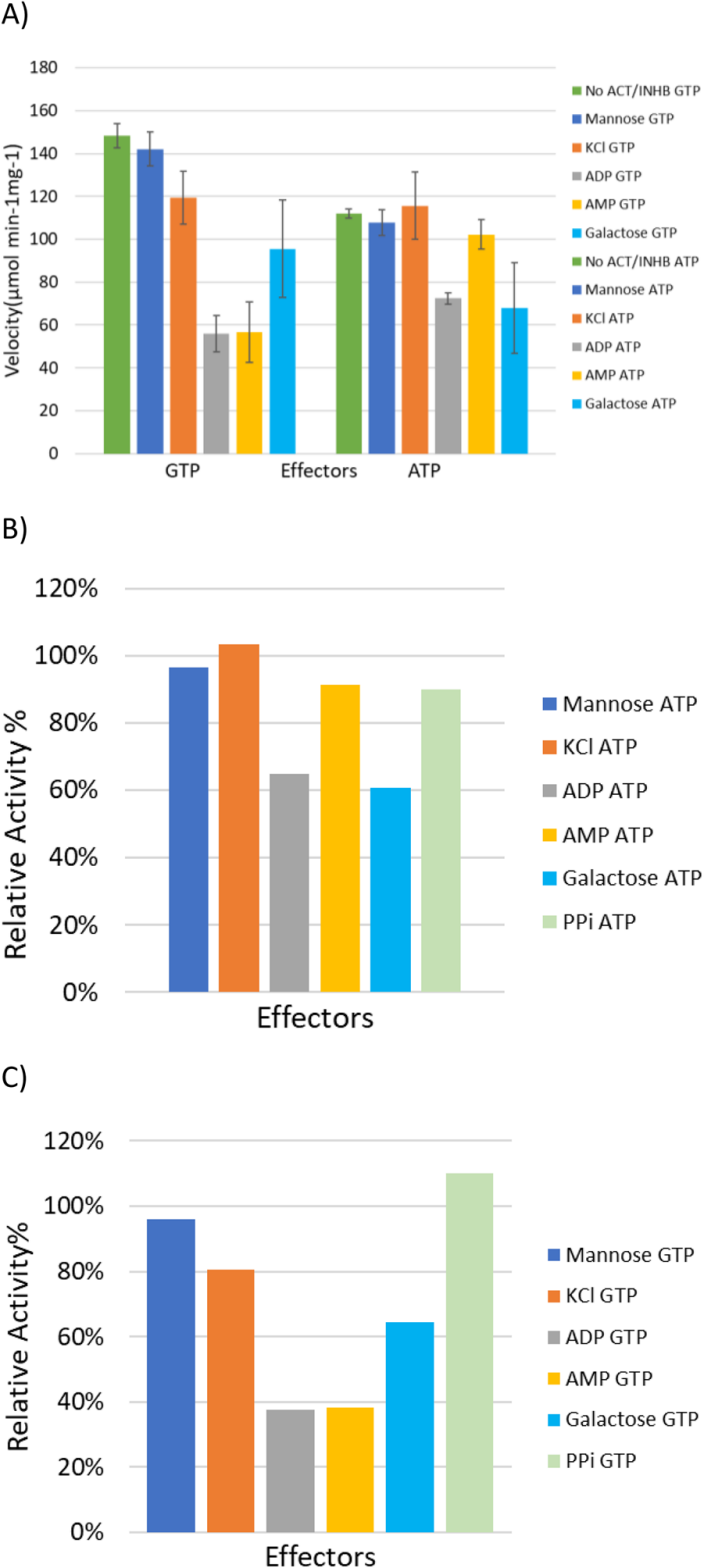
(**A**) Potential activating/ inhibiting compounds added to the assays to determine which conditions PfkB prefer as well as any possible feedback effect from other metabolic sugars. The velocity of the enzyme in each reaction was recorded in the presence of 2.24 mM substrate, 2.24 mM phosphate donor and 2.24 mM effector excluding MgCl_2_ (5.6 mM). (**B**) Relative activity of the enzyme with potential activating/inhibiting compounds added to the assays, compared to assays without any potential effectors other than MgCl_2_(5 mM) with ATP as the phosphate donor while (**C**) utilized GTP. The concentration of PP_i_ used in the assay was 0.4 mM, while the other effectors remained at 2.24 mM (**B**, **C**)

PfkB, unlike PfkA and other glycolytic enzymes such as malic enzyme is not inhibited by PP_i_ [9], therefore it could work in synch with Pfp (Fig. 8B, C). Out of all the effectors tested, it appears that AMP and ADP had the most significant effect on enzyme activity; observed as a sizeable decrease in relative activity when compared to the control reaction for both phosphate donors (Fig. 8). The inhibition was more prominent for reactions that utilized GTP as the phosphate donor, with activity being reduced to a mere 38% when either AMP or ADP (2.24 mM) was added to the assay, while ATP phosphorylated reaction decreased to 91% and 65% for AMP and ADP, respectively. Galactose also caused a noticeable decrease in activity, while K^+^ caused opposing result between phosphate donors. There was no statistical increase in activity when KCl was added to assays with ATP, but a significant (*p*-value < 0.05) decrease when added to reactions with GTP. *E. coli* ribokinase, a member of the PfkB family, can phosphorylate d-ribose and d-fructose among other monosaccharides and requires the presence both monovalent and divalent cation [48]. The maximum activity of the ribokinase was attained at a concentration of 5 mM MgCl_2_, while the optimum concentration of KCl was 0.1 M, but inhibition was observed above this concentration [48]. The recombinant PfkB of *C. thermocellum* does not follow this trend which could either represent an evolutionary change that alters the enzyme dependence on K^+^ or the concentration of KCl (2.24 mM) used in the assay was not optimum for proper activation.

#### Effects of pH on PfkB

**Fig. 9:**
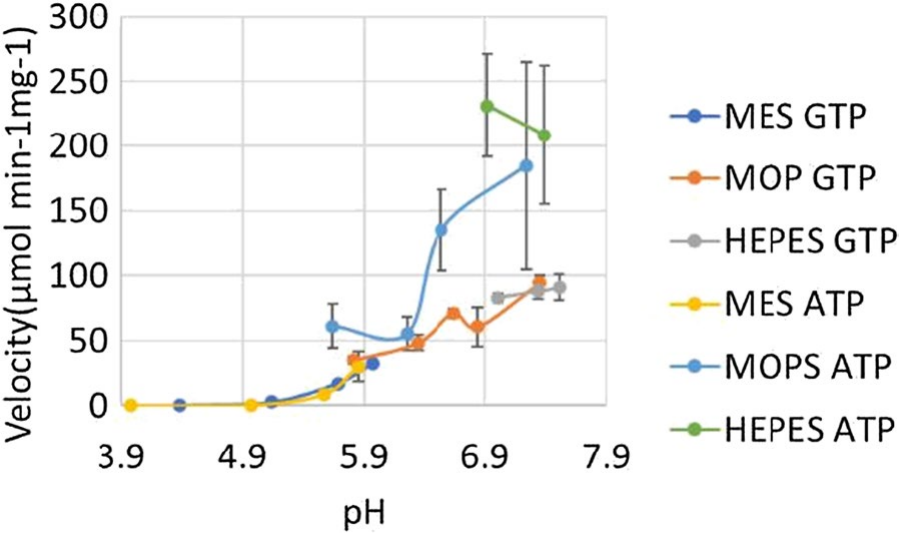
The pH range over which Pfkb showed activity with both phosphate donors and fructose as the substrate. To reduce confounding effects only sulfonic acid buffering agents with varying pK_a_ were used. The velocity of the enzyme in each reaction was recorded in the presence of 2 mM of the substrate and respective phosphate donor. Each assay conditioned was performed in triplicate

The optimum pH of PfkB appears to be 6.9 when ATP is utilized, while the GTP phosphorylation reactions appear to work better at pH 7.51 (Fig. 9). The pH range over which this enzyme is substantially active is narrower in comparison to the range of PfkA (Fig. 5), but their optima are still very close. This fact suggests that both enzymes could work in concert.

### Comparative structure–function relationship from primary sequence alignment of Family B PFKs

#### Substrate binding

**Fig. 10:**
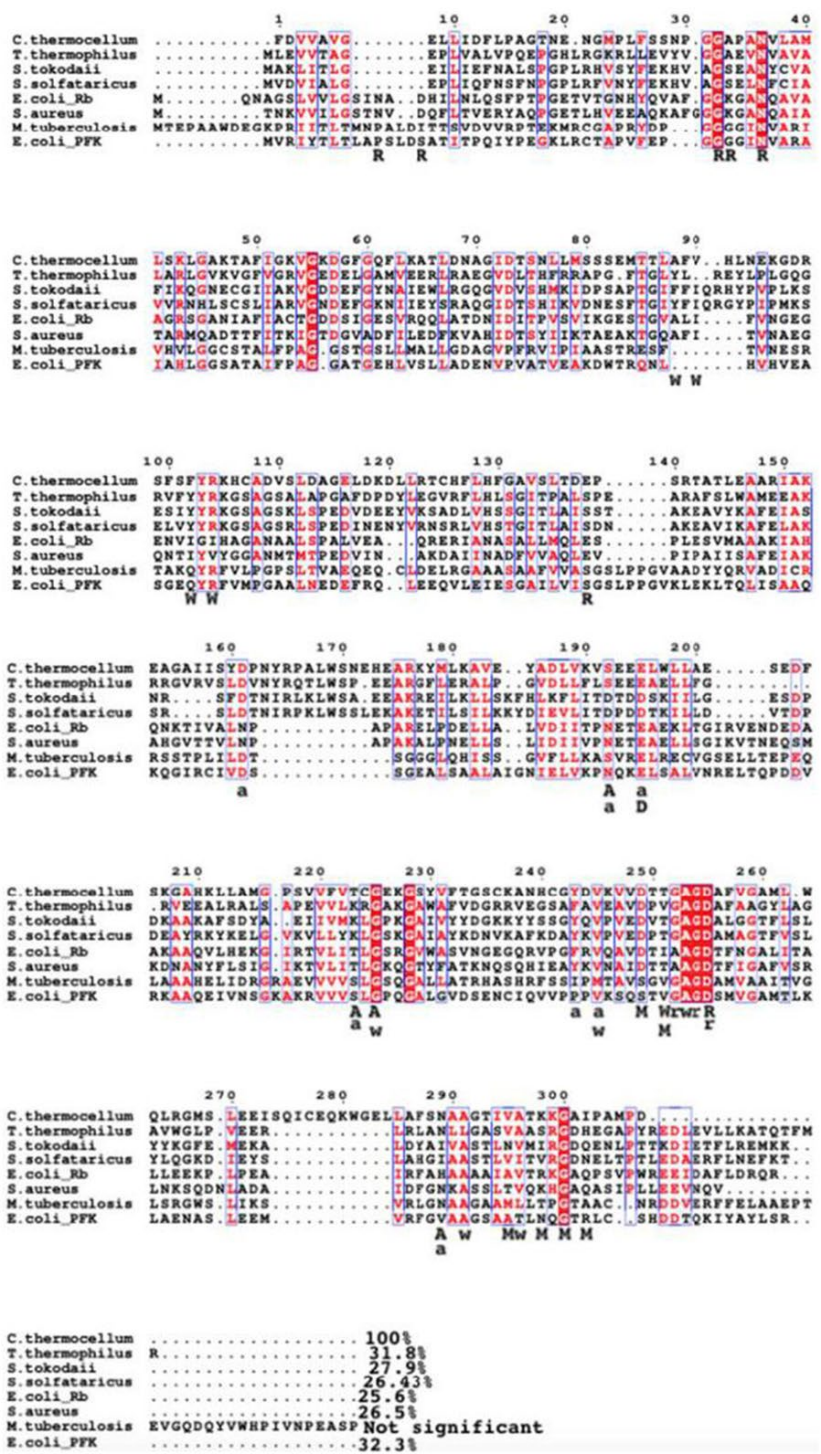
Amino acid alignments of Family B PFKs including the characterized ATP/GTP PfkB from *C. thermocellum;* along with the characterized PfkBs from a diverse group of microorganisms, which have been shown to utilize many different substrates. Protein crystal structures have been solved for *E. coli* ribokinase (*E. coli_*Rb*)*, which was used as a template to identify residues that directly hydrogen bond with ribose (**R**) and ATP (**A**), while indirect hydrogen bonds via water molecule with ribose (**r**) and ATP (**a**) was also shown. Residues from the protein that make van der Waals contacts with ribose (**W**) and ATP (**w**). The protein crystal structure of *E. coli* PFK-2(*E. coli_*PFK) was used to identify residues interacting with monovalent (**M**) and divalent cation (**D**). The percent similarity between C. *thermocellum* PfkB and each of the other PfkB were placed at the end of the alignment. Segments highlighted in red, identical residues, while letters in red represent similar residues. The sequences were aligned with MAFFT and the figure prepared with ESPript. The residues making up each of the PfkB single domain were used in the alignment

In comparison to members of the PfkA family, members of the PfkB family do not share the same level of residues conservation with respect to phosphate acceptor and phosphate donor binding (Fig. 10). This was expected in regard to binding the phosphate acceptor (substrate), as the PfkB family is made up of kinases such as adenosine, fructose, tagatose-6-P, fructose-6-P and ribose [49] (Table 4), but not with the residues implicated in binding the same phosphate donor. The PfkBs included in the alignment are displayed in Table 4, along with their phosphate acceptor, show the versatility of the kinases in this family in terms of phosphate accepting substrate. On a smaller scale, the ribokinase of *E. coli* (*E. coli_*Rb in the alignment) is able to utilize d-ribose, 2-deoxy-d-ribose, d-arabinose, d-xylose and d-fructose as phosphate acceptors [48].

**Table 4:** Characterized Pfks from two of the three phylogenetically distinct but related families

Organism	Phylum	$V\mathrm{max}(\frac{\mathrm{\mu mol}}{\mathrm{mg min}})$								*K*_m_ (mM)					Reference
Family	PfkA^l^														
*Geobacillus stearothermophilus*	Firmicutes, thermophile	–								F6P 0.033			ATP 0.07		[86]
*Thermotoga maritima*	Thermotogae, hyperthermophilic	F6P 464	ATP 432						GTP 294	F6P 0.437	ATP 0.009			GTP 1.36	[45]
*Thermus thermophilus*	Deinococcus-Thermus, extreme thermophile	–								F6P 0.025				ATP 0.08	[87]
*Streptomyces coelicolor A3(2)*	Actinobacteria, mesophile	F6P/ATP^a^ 165								F6P/ATP 1.0/0.4					[88]
*Mycobacterium tuberculosis H37Rv*	Actinobacteria, mesophile	F6P 20		ATP 19				T6P 30		F6P 0.4	ATP 1			T6P^J^ 0.43	[43]
*Amycolatopsis methanolica*	Actinobacteria, mesophilic or moderately thermophilic	F6P/ATP^m^ 167/180								F6P/ATP 6.0/0.6					[59]
*Lactobacillus delbrueckii subsp. bulgaricus*	Bacillota	F6P/ATP 90								F6P 0.3	ATP(pH 8.2) 0.2			ATP (pH 6.0)0.07	[58]
	Pfp^l^														
*Thermotoga maritima*	Thermotogae, hyperthermophilic	PP_i_ 203	PPP_i_ 249				Poly-P 319			PP_i_ 0.067		PPP_i_ 0.010		Poly-P 0.0038	[45]
*Methylococcus capsulatus*^*c*^	Proteobacteria, thermotolerant	F6P 7.6	S7P 31				FBP 9.0			F6P 2.27		S7P 0.03		PP_i_ .0.027	[41]
*Rhodospirillum rubrum*	Proteobacteria, mesophile	F6P 20			FBP 24.2					F6P 0.38		FBP 0.02		PP_i_ 0.025	[89]
*Xanthomonas campestris pv*	g-Proteobacteria, mesophile	F6P 58			FBP 59					F6P .202		FBP 0.024		PP_i_ .041	[90]
*Amycolatopsis methanolica*	Actinobacteria, mesophilic or moderately thermophilic	F6P 58				FBP 59				F6P 0.4	FBP 0.025			PPi 0.2	[91]
*Entamoeba histolytica*	Amoebozoa	–								F6P 0.038	S7P 0.064			PPi .014	[40, 92]
*Borrelia burgdorferi*	Spirochaetes	–								F6P .109				PPI .015	[93]
PfkB															
*Mycobacterium tuberculosis H37Rv*^*b*^	Actinobacteria, mesophile	F6P 1.2			T6P 1.5					F6P 0.04		T6P^J^ 0.09			[43]
*E. coli K12*	Proteobacteria, mesophile	–			–					F6P 0.006		ATP 0.008			[50]
*E. coli K12*	Proteobacteria, mesophile	D-Ribose^e^ 81			ATP^f^ 122					d-Ribose^e^ 0.279		ATP 0.213			[48]
*Staphylococcus aureus*	Firmicutes	–			–					ATP 4.66		ATP^d^ 2.19			[94]
*Thermus thermophilus*	Deinococcota	–			–					KDG^g^ 0.32		KG 3.6			[95]
*Sulfurisphaera tokodaii*	Crenarchaeota	KDG 4.13			KG 13.26					KDG^h^ 0.027		KG^i^ 1.3			[96]
*Saccharolobus solfataricus*	Crenarchaeota	KDG 9.7								KDG^k^(@ 70 °C) 0.14				ATP (@ 60 °C) 2.8	[97, 98]

^a^Michaelis–Menten kinetics displayed with ATP(1 mM) and varying concentration of F6P up to 10 mM

^b^This Pfk B was also able to catalyze the gluconeogenic reaction with FBP, ADP, IDP and GDP as substrates

^c^The *K*_m_ of phosphate and diphosphate are 8.69 mM and 0.027 mM, respectively

^d^In the presence of 100 mM K^+^

^e^Recombinant enzyme also able to utilize 2-deoxy-d-ribose, d-arabinose, d-xylose and d-fructose as substrate but less efficiently. *K*_m_ of d-ribose also decreases with the addition of pentavalent ions (phosphate, vanadate, arsenate), as well as increased *V*_max_

^f^GTP was also a phosphate donor

^g^2-Keto-3-deoxygluconate (KDG) & 2-keto-d-gluconate (KG) were the substrates under investigation. Activity recorder as *k*_cat_ for each substrate was excluded from the table for comparative reasons with *V*_max_

^h^*K*_m_ of ATP with KDG is 0.036

^i^*K*_m_ of ATP with KG is 0.11

^J^K_0.5_ rather than *K*_m_, as kinetic data were fitted to a sigmoidal (Hill) response curve

^k^*K*_m_ of KDG increases to 3.6 mM at 60 °C. 2-keto-3-deoxygalactonate is also a substrate, but affinity is twofold lower than KDG under similar conditions

^l^PfkA and Pfp both belong to Family A PFK

^m^*V*_max_ of PfkA when ATP concentration held constant at 1 mM while [F6P] varied, followed by *V*_max_ when [F6P] held constant at 10 mM while [ATP] varied

The PfkB of *C. thermocellum,* characterized with fructose (in its pyranose form) does share some identical residues with the ribokinase of *E. coli* involved in making direct hydrogen bonds with the pyranose form of ribose. These identical residues include Gly32, Asn36, Glu138 and Asp255. Gly32 and Asn36 are known to be a part of 1 of 2 motifs seen in ribokinases [24]. Identifying these residues in *C. thermocellum* PfkB is not an indication that the enzyme is able to utilize ribose, as this motif includes 15 residues, all of which are not identical between *E. coli* ribokinase and *C. thermocellum* PfkB. The second motif seen in ribokinases includes Asp255, which acts as a catalytic base, encouraging deprotonation of the sugar hydroxyl for a nucleophilic attack [24] in a similar fashion to Family A PFKs as previously mentioned. The residues around the aspartic acid from Family A and B PFKs are completely different, in addition to the lack of sequence conservation throughout the alignment. These findings suggest that the enzymes evolved convergently, acquiring the aspartic acid residue to perform the same function. However, the phylogenetic tree constructed (see Additional file 5) depicts Family A and B rooted on the same tree, with divergence between the families occurring closer to the root, supported by UFBoot values ≥ 95. This provides evidence that *C. thermocellum pfkb* once shared a common very distant ancestor with either *pfkA* or *pfp* before a gene duplication event occurred (see Additional file 5).

### Interaction with phosphate donors

The interaction with the phosphate donor is facilitated by direct hydrogen bonds, indirect hydrogen bonds via water molecules and Van der Waals interactions [24]. These interactions were determined for *E. coli* ribokinase, but both identical or similar residues appear in other members of the PfkB family at a higher percentage of conservation in comparison to interactions with the phosphate acceptor. The second motif observed from position 245–261 displays the strongest level of conservation among enzymes (Fig. 10) and is known for binding the adenine ring of ATP; in addition to forming the anion hole, required for stabilizing the deprotonated Asp255 [24].

As mentioned above, addition of K^+^ to the fructokinase assays caused a decrease activity when GTP is phosphate donor, which contradicts what is seen in many ribokinase, that require a monovalent cation in order to help shape the anion hole [24, 48, 50, 51]. The modest inhibition observed for *C. thermocellum* PfkB is in agreement with the inhibition observed for *E*. *coli* minor Pfk-2 [50], but to a lesser extent. It is proposed that the K^+^ is not an inhibitor but promotes the allosteric binding of MgATP, which acts both a substrate and an allosteric effector [50]. The presence of both MgATP binding sites in *C. thermocellum* PfkB was not determined, but there was very little conservation observed among the residues implicated in binding the monovalent cation between *E*. *coli* minor Pfk-2 and *C. thermocellum* PfkB (Fig. 10). This is one possible reason as to why inhibition was not as definite for *C. thermocellum* PfkB as the concentration of K^+^(2.24 mM) falls within the inhibitory concentration range (2-5 mM) observed for *E*. *coli* Pfk-2 [50]. The other reason is associated with the concentration of Mg^2+^ and ATP (forming MgATP species), which is the proposed substrate and effector. The initial concentration of Mg^2+^ was 5.6 mM, while ATP was 2 mM, thus there would be free Mg^2+^ ions after the formation of the MgATP effector, which has been shown to attenuate the inhibition of MgATP by potentially competing for the allosteric binding site [49]. This regulation is observed in *E*. *coli* minor Pfk-2 which relies on Glu195 (comprising a conserved NXXE motif) in the active site to bind the catalytic Mg^2+^, also seen in *C. thermocellum* PfkB (Fig. 10) [49]. So even if *C. thermocellum* PfkB has the ability to bind K^+^, to increase the affinity for the inhibitory MgATP species, the excess Mg^2+^ ions would negate this effect.

### Purification of the FBP aldolase

The monomeric molecular mass of the cloned and purified His-tagged Aldoa was approximately 36 kDa based off the SDS-PAGE, which is in close agreement with the molecular mass derived from the amino acid sequence (33.6 kDa), in addition to the ~ 4-kDa linker and N-terminal histidine tag. This observation, as well as the mass spectra analysis of the actual tryptic digest compared to the theoretical digest of the cloned sequence, confirms both the identity of the protein and purity of the elution.

### Kinetics of Aldoa

The cleavage reaction catalyzed by Aldoa was characterized by varying the concentration of the substrate, fructose 1,6-bisphosphate. Kinetic parameters were determined by fitting the data to the Michaelis–Menten equation using Sigma-Plot. Based on the analysis, the *K*_m_ and *V*_max_ of FBP are 0.145 mM and 27.7 μmol/mg/min, respectively (see Additional file 6: Table S10). There are two aldolase gene encoded in the genome of *C. thermocellum,* Cthe_0319 and Cthe _0349. The latter of the two, which is being characterized in this study, was shown to be highly expressed in the proteome in concert with the characterized Pfp [13]. The *V*_max_ of Aldoa and Pfp (Table 2) are in better agreement in comparison to PfkA (Table 1), which is consistent with their high expression.

### Effects of metal ions on Aldoa activity

**Fig. 11:**
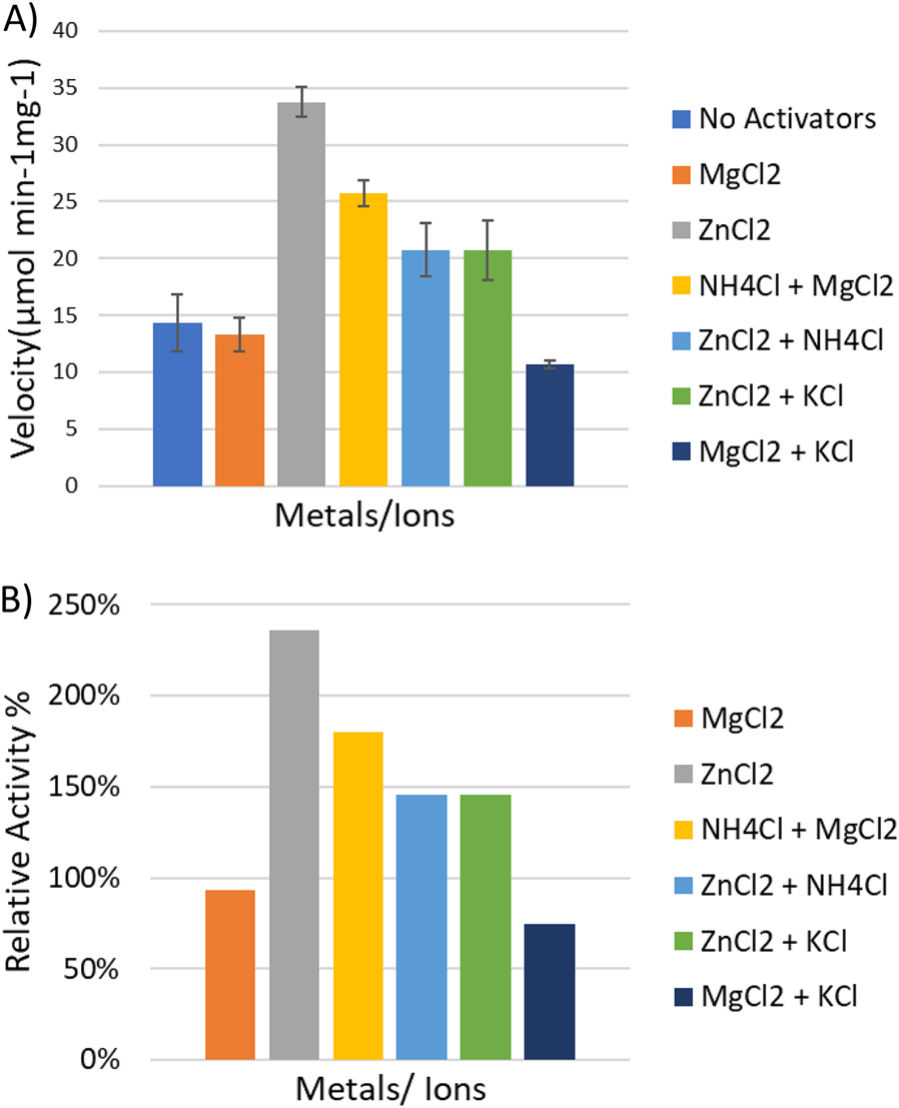
(**A**) Effect of metal ions on Cthe_0349, aldolase activity. Kinetic assays were performed with various divalent and or monovalent cations at a final concentration of 2 mM at 55 °C. Bars represent means ± standard deviations for 3 replicates. (**B**) Effects of metal ions on the relative activity of Cthe_0349 in comparison to assays in which no metal ions were added. All subsequent assay for the characterization of Cthe_0349 was done with MgCl_2_ and NH_4_Cl

*Clostridium thermocellum* aldolase is a member of the class II (type B) FBP aldolases, which are metalloenzymes and require a divalent metal ion [27]. This is confirmed by assays done with Zn^2+^, in which a > twofold increase in activity was observed when compared to assays done without metal ions (Fig. 11) (see Additional file 6: Table S11). The enzyme appears to have a preference regarding which divalent ion is bound, as addition of Mg^2+^ does not cause a significant change in activity when compared to the control (Fig. 11A). However, a combination Mg^2+^ and NH_4_^+^ did result in a significant increase in activity compared to the control. This confirms the findings in previous literature, that class II aldolases require a monovalent cation for maximal activity [26, 29, 52].

There is a caveat for monovalent activation in terms of K^+^, which causes a decrease in activity when utilized with either Zn^2+^ or Mg^2+^; whereas the addition of NH_4_^+^ to assays with Zn^2+^ does attenuate activity when compared to samples with solely Zn^2+^. However, the latter combination still produces an increase in activity relative to the control. These findings, along with the amino acid sequence alignment (see Additional file 4) that reveal conserved residues implicated in binding divalent and monovalent metal ions, validate that *C. thermocellum* Aldoa belongs to class II (type B). Class I FBP aldolases do not require divalent metal ions. Instead, they contain a lysine residue in the active site which forms a Schiff base intermediate with the substrate [30, 53].

### Effects of pH on Aldoa

**Fig. 12:**
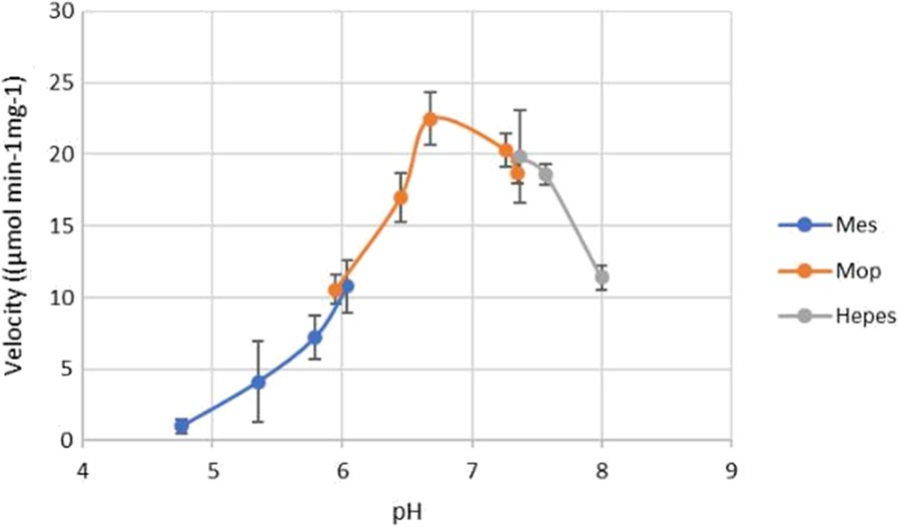
The pH range over which Aldoa showed activity. In order to reduce confounding effects only sulfonic acid buffering agents with varying pK_a_ were used. The velocity of the enzyme in each reaction was recorded in the presence of 2 mM of the substrate. Each assay condition was performed in triplicate

The optimal pH of Aldoa appears to be 6.68, with an immediate fall off in activity observed on either side of the optimum pH (Fig. 12). This narrow optimal pH range is a characteristic seen in other class II FBP aldolases [54, 55], while class I FBP aldolases have a broad optimal pH range (pH 7.0–9.0) [56].

### Phylogenetic analysis of PFKs

Phylogenetic analysis revealed the three PFKs under investigation can be separated into three distinctive clades (see Additional file 5). The probability of the clade being true was assessed by ultrafast bootstrap values (≥ 95%) [57]. The PfkA clade was well supported in comparison to PfP and PfkB, which is due to the fact that enzymes belonging to this group show the least amount of variability in terms of the substrate to be phosphorylated (Table 4). Enzymes belonging to Family A (PfkA and Pfp) were closer in relation, exemplified by the location of *A. methanolica* Pfp lodged in the middle of the PfkA clade compared to Family B Pfks (see Additional file 5). All Family B PFKs grouped together on a completely different branch, thus the split from Family A Pfks must have occurred in advance of the divergence between PfkA and Pfp.

Sequence alignments (Figs. 7 and 10) displaying the catalytic base (aspartic acid) as well as surrounding residues suggest convergent evolution between Family A and Family B Pfks; rather than their presence in their common ancestor. The actual evolutionary mechanism goes beyond the scope of the paper; but the consensus that both families are phylogenetically distinct remains.

*Clostridium thermocellum* PfkA was closer to both *Lactobacillus delbrueckii subsp. Bulgaricus* and *Geobacillus stearothermophilus* among characterize Pfks included in the analysis. Like *C. thermocellum* PfkA, *L. bulgaricus* PfkA is also insensitive to ADP, while the dinucleotide is an activator for *G. stearothermophilus* PfkA. The initial difference that emerges while comparing the bacteria physiologies is that the latter organism is aerobic, while the former two are strict anaerobes. It has been suggested that the lack of control of Pfks from anaerobes by ADP, corresponds to the lack of regulation by oxygen on glycolysis via the Pasteur effect [58]. *Thermotoga maritima,* an anerobic bacteria, a part of the same clade contradicts the previous notion in regard to PfkA activation by ADP [45]. It has been reported that hyperthermophilic bacteria usually rely on ADP rather than ATP due to ADP being slightly more thermostable compared to ATP [20]. Characterization of *T. maritima* PfkA determined ATP among other triphosphates to be the phosphate donor [45], but the hyperthermophilic nature of the bacteria may have caused its metabolic enzymes to evolve differently in the presence of ADP compared to mesophilic and thermophilic anaerobes. *T. maritima* encodes both a PfkA and Pfp, much like *C. thermocellum;* in addition to PfkA being inhibited by PP_i_ and polyphosphate. *T. maritima* appears to express both isoforms of the enzyme simultaneously, but activity has been proven to be dependent on the pH [45], while it is assumed that PP_i_ or polyphosphate may also play a similar controlling role. Similar conditional expression between Pfp and PfkA was also observed in *A. methanolica* [59]. These findings give support to the case of C. *thermocellum* being able to express both Pfp and PfkA under the right condition to fulfill different physiological roles.

## Discussion

The *pfkA* encoded by *C. thermocellum*, although not highly expressed in the proteome of cellobiose grown cells [13], is capable of substantial activity with F6P as the substrate and either ATP or GTP as the phosphate donor. Compared to the highly expressed Pfp, PfkA produces FBP at a faster rate and only catalyze the forward reaction. Pfp ability to catalyze both the forward and reverse reaction was revealed to be a key factor reducing the thermodynamic driving force in the upper glycolysis pathway [15]. The reversible reaction affords *C. thermocellum* the flexibility to perform either glycolysis or gluconeogenesis [15].The ability to transition between either pathway may confer the bacteria certain advantages, required for growth in its environmental setting. However, this flexibility appears as a disadvantage in an industrial setting aimed at optimizing ethanol production, linked to glycolysis. The significance of Pfp reversibility becomes apparent after kinetic assays show the enzyme ability to phosphorylate S7P.

*Clostridium thermocellum* does not have a complete pentose phosphate pathway as it lacks genes encoding glucose-6-phosphate dehydrogenase, gluconolactonase and 6-phosphogluconate dehydrogenase in the oxidative branch required for generating NADPH for biosynthesis; as well as the transaldolase in the non-oxidative branch used to produce ribose-5-phosphate (R5P) and erythrose-4-phosphate required for nucleotide and aromatic amino acid synthesis, respectively [13, 21]. *C. thermocellum* may be able to compensate for the missing transaldolase in a similar fashion as seen in *Entamoeba histolytica* [40, 60, 61]*.* In order to produce pentose intermediates, it has been proposed that the FBP aldolase (Aldoa) convert dihydroxyacetone-P and erythrose-4-P into SBP, previously shown by Susskind et al. Followed by the dephosphorylation of SBP to S7P by the Pfp under investigation, which will subsequently be converted to R5P and xylulose-5-phosphate by the transketolase performing the carbon transfer reaction between S7P and glyceraldehyde-3-P (Fig. 1). Although the current study only tested S7P phosphorylation, the low ΔG associated with PP_i_ phosphorylated reactions, allows both the forward and reverse reactions [15]. With this knowledge taken into consideration, Pfp cannot simply be replaced with a higher velocity ATP-PFK, which lacks the ability to dephosphorylate SBP. The metabolic flexibility afforded by Pfp also extends to engineered strains of *C. thermocellum*, modified to grow on xylose [62]. The introduction of both xylose isomerase and xylulokinase into *C.* *thermocellum* genome allows for the metabolism of xylose to xylulose-5-phosphate; which is further metabolized through the pentose phosphate pathway relying on the conversion S7P to SBP by Pfp to confer growth (Fig. 1). This apparent lack of transaldolase appears to be common in other more closely related lignocellulolytic clostridia such as *Clostridium stercorarium*, *Clostridium termitidis* or *Clostridium thermosuccinogenes* [63–65].

Co-expression of both Pfp and PfkA is one plausible solution to attain proper growth, while alleviating upper glycolysis bottleneck caused by the slower Pfp reaction. A fault in this hypothesis arises when considering the conditions under which the current PfkA encoded by *C. thermocellum* is active. PfkA is inhibited by PP_i_ concentrations as low as 0.05 mM (Fig. 4D), thus it cannot be said with confidence that both PfP and PfkA could be active at the same time. Pfp reaction in the altered pentose phosphate pathway (Fig. 1) would be producing PP_i_ rather than utilizing it, thus as long as the concentration PP_i_ remains low, then the PfkA reaction should be possible. Lower PP_i_ concentrations would also have positive implications on the malic enzyme (MalE) activity, participating in the malate shunt pathway; which is responsible for producing GTP (phosphate donor of PfkA), transferring electrons between NADH and NADP^+^ and converting PEP, inhibitor of PfkA, into pyruvate [9, 10].

Pyrophosphate metabolism in *C. thermocellum* is quite diverse, involving metabolic reactions responsible for either the generation or hydrolysis of PP_i_. Reactions that actively generate PP_i_ include the modified pentose phosphate pathway via the PP_i_-dependent PFK (Pfp), glycogen cycling, translocation of H^+^ coupled to either PP_i_ hydrolysis or synthesis via a membrane-bound pyrophosphatase and the Ppdk-malate shunt cycle [66–70]. Consumption of PP_i_ may occur through various glycolytic reactions catalyzed by Pfp, pyruvate phosphate dikinase (PPDK) and acetate thiokinase [10].

The FBP produced by the PFK reaction will subsequently be cleaved by the fructose-1,6-bisphosphate aldolase (Aldoa) into dihydroxyacetone-phosphate (DHAP) and glyceraldehyde 3-phosphate (G3P) in the glycolysis pathway. Characterization of Aldoa has revealed that this reaction is partially responsible for the kinetic bottleneck observed in the upper glycolysis pathway, causing the accumulation of FBP [15, 16], because of its slow velocity in vitro. The initial glycolysis reaction catalyzed by glucokinase, is able to utilize both ATP and GTP similar to PfkA, while boasting *V*_max_ values ranging from 128.9 µmol/min mg (with ATP) to 1023.2 µmol/min mg (with GTP) depending on the phosphate donor [37]. The glucose-6-phosphate isomerase has yet to be characterized; therefore, the PfP-catalyzed reaction is the first limiting reaction observed, followed by the Aldoa-catalyzed reaction, which both suffer from lower reaction velocities and operate close to thermodynamic equilibrium (low ΔG) [15].

The proposed alternative pentose phosphate pathway depends on the ability of Aldoa to convert erythrose-4-phosphate and DHAP into SBP before subsequent dephosphorylation by Pfp (Fig. 1). This proposed Aldoa-catalyzed reaction was not verified in the current study, but previous characterization of *Cyanophora paradoxa* class II aldolase has shown activity with SBP as a substrate [30, 31]. In order to produce substantial activity, Aldoa requires the presence of Zn^2+^ as the divalent metal ion, while NH_4_^+^ is also able to activate the enzyme. The latter activation with the monovalent ion is also common to PfkA, MalE and PPDK. These findings suggest that the glycolysis pathway is regulated by both NH_4_^+^ and PP_i_ at the protein level.

The characterized PfkB, determined to be a fructokinase, is not expected to take part in the glycolysis pathway for cellobiose or Avicel-grown cells. This assumption is supported by the low level of expression observed in the proteome of cellobiose grown cells [10]; as well as the fact that catabolism of either cellobiose or Avicel via glycolysis, does not produce fructose as an intermediate. Thus, there is no substrate in the current proposed glycolysis pathway that the enzyme can act on. An increase in fructokinase activity from cell-free extract have been observed in fructose-grown cells (*C.* *thermocellum* DSM 1313) [47]; while preliminary qPCR results have shown an increase *pfkB* transcription in fructose-grown cells (C. *thermocellum* ATCC 27405). Unlike PfkA, Aldoa and other enzymes involved in glycolysis, PfkB activity is neither affected by NH_4_^+^ or PP_i_. Instead, galactose, AMP and ADP were observed to be the main inhibitory effectors of the enzyme. Feedback inhibition appears to be one of the key factors controlling the activity of the enzyme; therefore, it is possible that PfkB is linked to galactose metabolism, or a connected metabolic pathway.

## Conclusion

Kinetic characterization of Pfp, PfkA, PfkB and Aldoa have partially revealed their roles in *C.* *thermocellum* ATCC 27405 metabolism; as well as shed some light on the metabolic redundancy of the bacteria. Although enzymes have been shown or predicted to carry out multiple roles in the organism, there is still a high level of regulation occurring at the transcription and protein level for various enzymes in the glycolysis pathway, which may vindicate the organism’s redundancy. Phylogenetic analysis suggests that these proteins have evolved to fit their biological function(s) which is concurrently determined by the conditions under which the bacteria grows and metabolic capabilities including metabolite pool. These properties grant *C.* *thermocellum* metabolic flexibility which would be advantageous for a bacterium isolated from lignocellulose containing environments, that is known to thrive in a consortium with other microbes.

Resolving the thermodynamic bottleneck introduced by Pfp and Aldoa would be a step in the right direction for increased biochemical production, but gene replacement may cause adverse effects if *C.* *thermocellum* is unable to account for the loss of the proposed alternative reactions catalyzed by these enzymes. Co-expression of a PfkA with genetic mutations to evade PP_i_ inhibition could be the solution to the upper glycolysis bottleneck, with FBP accumulation forcing the Aldoa reaction forward. Further understanding of the interwoven metabolism of C. *thermocellum* to reveal the players in carbon catabolism and redox balancing would aid in designing a suitable pathway for the production of any biochemical in *C. thermocellum.*

## Materials and methods

### Strains and reagents

The genomic information from the type strain of *Clostridium thermocellum,* DSM 1237, was used throughout these experiments as the source for the proteins expressed. All gene locus tags described refer to this strain. *Escherichia coli* DH5α was used as the host for plasmid screening, while *E*. *coli* T7 Shuffle (New England Biolabs) was used as the expression strain for recombinant protein expression. Plasmids were extracted using the Qiagen QuickLyse Miniprep Kit (Qiagen Inc.) All PCRs were done using the DreamTaq PCR Master Mix(2x) (Thermo Scientific™). PCR purification was carried out using the ChargeSwitch® PCR Clean-Up Kit (Invitrogen™ by Life Technologies™). All restriction enzymes used were purchased from New England Biolabs. Recombinant proteins were purified using HiTrap™ chelating HP column (GE Healthcare Bio-Sciences Corp.), followed by dialysis with SnakeSkin™ Dialysis tubing 10,000 MWCO (Thermo Scientific). Protein gels for subsequent protein identification was made using TGX FastCast Acrylamide Kit, 10% (Bio-Rad). All antibiotics used were purchased from Sigma-Aldrich. Fructose-6-phosphate (F6P), fructose-1,6-bisphosphate (FBP), fructose, phosphoenolpyruvate (PEP), d-tagatose-6-phosphate (T6P), glucose-6-phosphate(G6P), malic acid, mannose, inosine, NADP^+^, NADPH, NAD^+^, NADH, ATP, ADP, AMP, GTP, pyrophosphate (PP_i_), all coupling enzymes; as well as buffers used were from Sigma-Aldrich. d-Sedoheptulose-7-phosphate (S7P), citric acid and galactose were obtained from Carbosynth, J.T.Baker chemical co. and Fisher Scientific Company, respectively.

### Plasmid preparation

Plasmids were designed using the corresponding gene sequences from C. *thermocellum* ATCC 27405 retrieved from Integrated Microbial Genomics [71] for *pfkA* (Cthe_1261), *pfkB* (Cthe_0389) and FBA (*aldoa*) (Cthe_0349), which were codon optimized for expression in *E. coli*. The plasmids were synthesized by Geneart® (Life Technology Inc. Burlington, Ontario) using a pRSET-A backbone to form the recombinant plasmids pAHCT1261 (*pfkA*), pAHCT0389 (*pfkB*) and pAHCT0349 (*aldoa*), each containing the gene of interest. pAHCT0347(*pfp*) was constructed and cloned into *E*. *coli* T7 Shuffle from a previous study. The genes were inserted downstream of a T7-inducible promoter and the His_6_-tag sequence, located at the N terminus of the recombinant plasmid. *E. coli* DH5α was used as the initial transformation host, followed by isolation of the plasmid using the Qiagen QuickLyse Miniprep Kit, which was subsequently transformed into *E*. *coli* T7 Shuffle for recombinant protein expression following verification of the plasmid. Both *E. coli* strains were initially made competent for transformation, then transformed with respective plasmids following protocols adapted from New England Biolabs for both procedures.

### Verification of transformation

Plasmid DNA extracted from isolated colonies using the Qiagen QuickLyse Miniprep Kit was verified using restriction digest. HindIII, XhoI and EcoRI, were used in the restriction digest protocol adapted from New England Biolabs. PCR of the restriction digest was carried out using the T7 promoter sense primer 5′-TAATACGACTCACTATAGGG-3′ and the T7 terminator antisense primer 5′-GCTAGTTATTGCTCAGCGG-3′ (Alpha DNA), following the protocols included with the DreamTaq PCR™ MasterMix(2x), containing all the necessary reagents. The resulting PCR product concentration was measured using Qubit™ Assay, followed by visualization of the amplified DNA bands on an agarose gel. Plasmid constructs were verified via sequencing following PCR clean up (Centre of Applied Genomics, The Hospital for Sick Children, Toronto, Ontario).

### Overexpression and recombinant protein isolation

*Escherichia* *coli* T7 Shuffle containing either pAHCT1261, pAHCT0389, pAHCT0347 or pAHCT0349 was cultured overnight in LB media containing ampicillin (100 μg/ml) at 37 °C. The overnight culture was then sub-cultured into fresh LB media with the required amount of ampicillin and grown at 37 °C until an optical density at 600 nm (OD_600_) of 0.5 to 0.7 was reached. Isopropyl β-d-1-thiogalactopyranoside (IPTG) was added to the culture at a final concentration of 0.44 mM to induce expression and incubated for overnight with shaking at 20 °C. Cells were harvested by centrifugation at 10,000 rpm for 30 min at 4 °C and resuspended in buffer containing 20 mM (3-(*N*-morpholino) propane-sulfonic acid (MOPS) (pH 7.4), 500 mM NaCl, 20 mM imidazole. Cells were lysed at 37 °C after 20 min of incubation with phenylmethylsulfonyl fluoride (PMSF), 5 mg/ml lysozyme, 1% Triton X-100, DNase and RNase, adapted from Maniatis et al. [72]. Additional lysis was carried out by sonic oscillation with a micro tip at 50% duty cycle, with an output limit no more than 1.5 using the Branson Sonifier 450 for six cycles, which includes 6 pulses with the sonicator followed by 30 s on ice. The cell lysate was cleared after being centrifuged at 16,000 rpm for 40 min at 4 °C and the supernatant collected. Supernatant for recombinant proteins, Pfp, PfkB and Aldoa were loaded onto a Ni^2+^ HiTrap metal affinity column, while PfkA was loaded onto a column charged with cobalt; both of which were previously equilibrated with binding buffer (20 mM MOPS, 500 mM NaCl, 20 mM imidazole (pH 7.4)). The recombinant His_6_-tagged proteins were eluted using a stepwise imidazole gradient. The eluted recombinant protein samples were dialyzed overnight into 20 mM MOPS and 20 mM NaCl (pH 7.4) using SnakeSkin™ Dialysis tubing 10,000 MWCO, in order to remove imidazole and excess NaCl. The purity of the enzyme sample was verified by SDS-PAGE with a 10% resolving gel and stacking gel, followed by staining of the recombinant protein with Coomassie Brilliant Blue R-250 for visualization. Protein concentration of pure samples was measured by Qubit™ Assay.

### Enzyme assays

All enzyme activities were measured in a 300 μL, 96-well plate with a total reaction volume of 200 μL at temperatures up to 55 °C, with the exception of reactions focused around Pfp performed at 50 °C for the purpose of comparisons. PfkA activity was measured in a standard reaction containing 100 mM MOPS (pH 7.5), 5 mM MgCl_2_, 10 mM dithiothreitol (DTT), 2 mM NH_4_Cl, 0.2 mM NADH (prepared in 10 mM MOPS buffer), 5U/ml aldolase, 5U/ml triosephosphate isomerase (TPI), 5U/ml glycerol-3-phosphate dehydrogenase (GDH). F6P, NH_4_Cl, ATP, ADP and GTP concentrations were varied, with ADP and NH_4_Cl playing a potential activator role. Prior to use, the coupling enzymes were desalted using a HiTrap™ desalting column and eluted in 20 mM MOPS and 20 mM NaCl (pH 7.4) in order to remove the ammonium sulfate that the enzymes were previously suspended in. Assays were started with the respective phosphate donor.

In order to verify that fructose was not a substrate of PfkA, the standard reaction mix outlined above was altered to remove F6P but add 2 mM fructose and PfkB as a control. Activity of PfkA was coupled to NADH oxidation tracked at 340 nm using SpectraMax® iD5. PfkB activity was measured in a reaction containing 100 mM MOPS (pH 7.5), 5 mM MgCl_2_, 10 mM DTT, 0.2 mM NADP^+^, 2.2 U/ml glucose-6-phosphate dehydrogenase (G6PD), 2 U/ml phosphoglucose isomerase (PGI). Fructose and phosphate donor (ATP & GTP) concentrations were varied. Assays were started with the respective phosphate donor. Activity of PfkB was coupled to NADP^+^ reduction tracked at 340 nm using SpectraMax® iD5.

Pfp activity was evaluated with both F6P and S7P, each requiring a separate assay. Probing F6P utilization by Pfp was measured in a standard reaction containing 100 mM MOPS (pH 7.5), 2.5 mM MgCl_2_, 10 mM DTT, 0.2 mM NADH, 5U/ml aldolase, 5U/ml TPI, 5U/ml GDH. F6P and PP_i_ concentration were varied. Assays were started with the sugar phosphate to avoid precipitation. Activity of Pfp was coupled to NADH oxidation as mentioned above. S7P utilization by Pfp was measured in a reaction adapted from Guillen Suarez et al. [73], which contains 100 mM MOPS (pH7.5), 2.5 mM MgCl_2_, 10 mM dithiothreitol (DTT), 0.25 mM inosine, 50 mU/ml purine nucleoside phosphorylase (PNPase), 500 mU/ml xanthine oxidase (XOD). S7P and PP_i_ concentration were varied. Assays were started with the sugar phosphate to avoid precipitation. Activity of Pfp was coupled to hypoxanthine oxidation, i.e., uric acid production, which can be monitored at 293 nm. In the case of this assay, the Greiner Bio-One 96-Well Non-treated Polystyrene Microplates, had to be substituted with Corning™ UV-Transparent microplates due to the fact that the polystyrene microplates were able to absorb at a wavelength of 293 nm. Competition assays with both sugar phosphates was thereafter ensued.

Aldoa activity was measured by coupling the cleavage of fructose-1,6-bisphosphate by the purified aldolase with the oxidation of NADH by GDH. The standard reaction mixture contained 100 mM MOPS (pH 7.5), 5 mM MgCl_2_, 10 mM DTT, 2 mM NH_4_Cl, 0.2 mM NADH, 5U/ml TPI, 5U/ml GDH. Assays were started by the addition of the substrate, fructose-1,6-bisphosphate, which was varied over the course of the experiment.

### Kinetic properties

Kinetic properties of PfkA, PfkB, Pfp and Aldoa were determined by varying the concentration of the respective sugar, sugar phosphate or phosphate donor, while keeping the concentration of all other components in the reaction mixture constant at saturated levels at 55 °C for PfkA, PfkB and Aldoa and 50 °C for Pfp, all at a pH of 7.5. Kinetic parameters were determined by fitting the data to the Michaelis–Menten equation using Sigma-Plot software (Systat Software Inc.).

### Effects of activators/inhibitors and cofactors

Potential activators or inhibitors of PfkA and PfkB including phosphate donors, glycolysis, and TCA cycle intermediates, metal ions and other sugar monomers were determined by adding the individual effector at a concentration of 2 mM to the standard reaction mixture outline above, while keeping the concentrations of other constituents constant, prior to taking activity measurements. Regarding PfkA inhibition assays with various concentrations of PP_i_ was carried out in the presence of 2.5 mM MgCl rather than 5 mM. The effects of both divalent and monovalent cations on Aldoa activity were determined by substituting the divalent and monovalent cation (Mg^2+^ or NH_4_^+^) in the standard reaction mixture with either 2 mM Zn^2+^ or 2 mM K^+^.

### Effects of pH

The pH range over which PfkA, PfkB and Aldoa were active was tested with different sulfonic acid buffering agents including: 2-morpholinoethanesulfonic acid (MES) (pH 3.98–6.08), MOPS (pH 5.64–7.54) and 2-[4-(2-Hydroxyethyl)piperazin-1-yl]ethane-1-sulfonic acid (HEPES) (pH 6.92–8) in order to determine the optimal pH for each enzyme. The pH of each buffer was calibrated at 55 °C, corresponding to the temperature of the assays.

### Analysis of protein

The eluted PfkB sample was run on an SDS-PAGE gel, stained with Coomassie Brilliant Blue R-250, was excised and placed in a microfuge tube containing 500 μL of nanopure water and stored for at least 24 h at 4 °C. In-gel digestion was carried out following the protocols outlined in Shevchenko et al. [74]. Pure eluted samples of PfkA and Aldoa were reduced with 10 mM DTT in 0.1 M NH_4_HCO_3_ for 1 h at 37 °C, followed by alkylation with 55 mM iodoacetamide in 0.1 M NH_4_HCO_3_ for 30 min, at room temperature, in the dark. The sample was dialysed into 0.1 M NH_4_HCO_3_ using Amicon® Ultra 10KDa MWCO filters (Millipore), followed by 3 washes to remove all other chemicals. The sample was transferred to a microfuge tube and incubated overnight at 37 °C with 0.01 mg/ml trypsin. Digested protein was desalted with a C18 SPE column following manufacturer’s instructions (Phenomenex, Torrance, CA, USA). The samples were eluted in 80% acetonitrile + 0.1% TFA, mixed 1:1 v:v with DHB matrix (saturated solution of 2,5-dihydroxybenzoic acid in 70:30 water: acetonitrile + 0.1% TFA), and applied onto a MALDI target. Mass spectra were acquired on a MALDI-TOF-TOF UltrafleXtreme (Bruker Daltonics, Germany) and analyzed using the Compass 1.4 FLEX series Bruker software. Some ions were further analyzed by tandem MS(MS/MS) using Bruker Lift™ technology in order to verify the exact sequence match. Spectra for each protein were compared to a theoretical tryptic digest of the cloned sequence created from Expasy PeptideMass [75, 76].

### Analysis of sugar phosphate

The sugar phosphate products of PfkA, Pfp and Pfkb were verified through mass spectrometry. Individual protein samples were dialysed into 10 mM NH_4_OAc at pH 7.4 using a 10 kDa MWCO spin column prior to carrying out the reaction with their respective sugar phosphates. Pfp reaction included 5 mM NH_4_OAc, 2.5 mM MgCl_2_, 10 mM DTT, 2 mM PP_i_ and 2.115 mM S7P, which was incubated at 50 °C for 15 min. After the elapsed time, each the reaction mixture was transferred to a 10 kDa MWCO spin column to remove the protein. The eluates were transferred to microfuge tubes and dried in a speed-vac to remove the volatile buffer, followed by resuspension in 30 μL of acetonitrile. ESI–MS analysis was carried out by direct infusion using autosampler, injecting an aliquot onto the ESI. Ionization mode was carried out in negative mode with TOF mass range calibrated from 50 to 2000 m/z (Elute SP autosampler, ESI-Quadrupole Time-of-Flight COMPACT, Compass 3.0 for OTOF series software, Bruker Daltonics, Germany). The measured masses were compared to the molecular weights of both S7P and its bisphosphate counterpart, sedoheptulose,1-7,bisphosphate to determine if the expected reaction took place. The same procedure was carried out for both PfkA and PfkB with the caveat that both F6P and S7P were individually tested as substrates for the enzymes.

### Phylogenetic analysis

Homologous protein sequences for each of C. *thermocellum* ATCC 27405 PFKs, PfkA, PfkB and Pfp, were curated using the consurf server, which used the HMMER algorithm to search the non-redundant sequence database for 200 sequences with the E-value cut off set to 0.0001 [77–79]. An alignment of all PFK sequences was constructed using MAFFT with L-INS-I selected as the alignment method [80, 81] and visualized with ESPript [82]. Additional PFK sequences were added to the dataset during alignment from the Database of Aligned Structural Homologs (DASH), providing homologous sequences for which the protein structures have being solved, with or without the substrate of the protein.

The programs supplied in IQ-TREE were used to analyze the phylogenetic relationships among the PFK proteins. The evolutionary history of the PFKs were inferred by using the maximum likelihood (ML) method based on the LG + F + G4 model of substitution [83, 84]. The consensus tree with a higher likelihood (− 184,147.1570) was used in comparison to the ML tree that was initially found. The reliability of each branch was assessed with ultrafast bootstrap approximation (UFBoot) (1000 replicates); as well as the single branch test, SH-like approximate likelihood ratio test (SH-aLRT) (1000 replicates) [57]. iTol was used to visualize and annotate the consensus tree produced using IQ-TREE [85].

## Supplementary Information

**Additional file 1.** Mass spectrometry showing phosphorylated products observed with PfkA. **Figure S1**. The enzyme was able to phosphorylate fructose-6-phosphate(F6P), which has a theoretical molecular mass of 259.81 g/mol to Fructose-1,6-bisphosphate (MW = 340.116 g/mol), while utilizing GTP as the phosphate donor. The starting substrate(F6P) was low in relative abundance thus the peak at 259.0223 m/z was not seen on the chromatogram but was recorded in the spectrum deconvolution report. The peak for Fructose-1,6-bisphosphate (FBP) was seen at 338.989 m/z. **Figure S2.** The enzyme was not able to utilize sedoheptulose-7-phosphate (S7P, MW = 290.162 g/mol) as a substrate. The chromatogram only displays a peak for S7P, while there was no sign of sedoheptulose-1,7-bisphosphate (SBP,MW = 370.14 g/mol) on the chromatogram or spectrum deconvolution report.

**Additional file 2.** FMichaelis–Menten kinetics of Pfp generated using SigmaPlot® 11.2 with data used to generate kinetic parameters and Pfp competitive assay. **Figure S3.** Activity of Pfp versus varied F6P concentration predicts a relatively low *K*_m_ of 0.075 mM for F6P, but high *V*_max_, while PP_i_(2 mM) is used as a phosphate donor. **Table S1.** Data used to generate kinetics parameters for Pfp utilizing F6P + PP_i_ in this study, Table 2. **Figure S4.** S7P is the substrate under investigation which is observed to have high *K*_m_ of 3.236 and a much lower *V*_max_ compared to assays with F6P. **Table S2.** Data used to generate kinetics parameters for Pfp utilizing S7P + PP_i_ in this study, Table 2. **Table S3.** The rate of FBP production by Pfp at 45 °C was determined with F6P alone and in the presence of equimolar amounts of both F6P & S7P. There was approximately a 75% decrease in the rate of FBP production when both substrates were used in comparison to when F6P was the sole substrate, indicating that S7P could be competing for the active site. The velocity for the phosphorylation of solely S7P (2.12 mM) is also included in the figure for comparison to F6P (2 mM) phosphorylation. The production of Sedoheptulose-1,7-bisphosphate was confirmed by Mass Spectrometry

**Additional file 3.** Mass spectrometry analysis of sugar phosphates present after the reaction with PfkB, being the phosphorylating enzyme in question. **Figure S5.** F6P, the starting substrate is the only sugar phosphate that can be identified on the chromatogram, thus FBP was not produced. **Figure S6.** S7H, the starting substrate is the only sugar phosphate that can be identified on the chromatogram, thus SBP was not produced. Both reactions were carried out with ATP as the phosphate donor.

**Additional file 4.** Amino acid alignments of class II FBP aldolases. **Figure S7.** Including the characterized FBP aldolase of *C. thermocellum* currently under investigation; as well as the other annotated FBP aldolase encoded by *C. thermocellum* (*C. thermocellum_*hyp). *Thermus aquaticus, Thermotoga maritima, Giardia lamblia, Helicobacter pylori, Geobacillus stearothermophilus* all belong to class IIB, while *E. coli, Corynebacterium glutamicum, Saccharomyces cerevisiae, Euglena gracilis* belong to class IIA. The conserved residues implicated in binding the Zn^2+^ (**D**) and monovalent cation (**M**) have been identified based off stucturalr studies done the aldolase of *E. coli* [52]. Segments highlighted in black, identical residues, while letters that are bold represent similar residues. The sequences were aligned with MAFFT and the figure prepared with ESPript. The percent similarity between C. *thermocellum* characterized Aldoa and each of the other aldolases were placed at the end of the alignment.

**Additional file 5.** Phylogeny tree of PFK family. **Figure S8**. Phylogenetic analysis of 621 PFK sequences belonging Family A and B under the broader PFK superfamily by the maximum likelihood method. Characterized PfkA from different organisms were highlighted in red, Pfp highlighted in green and PfkB highlighted in purple. The tree is unrooted but *Intestinirhabdus alba* was the outgroup taxon drawn at the root. The tree is drawn to scale, with branch lengths measured in the number of substitutions per site. The probability of the branch being true was assessed by ultrafast bootstrap approximation, with values ≥ 95% considered reliable.

**Additional file 6:** PfkA, PfkB and Aldoa kinetic data used to generate kinetic parameters in this study. **Table S4.** Velocities produced by PfkA while varying the concentration of either ATP or GTP to determine the *K*_m_ of the phosphate donors while F6P was held at a concentration of 2 mM. Values generated kinetic parameters in Table 1. **Table S5.** Velocities produced by PfkA while varying the concentration of F6P to determine the *K*_m_ of the sugar phosphate while phosphate donor (ATP/GTP) concentration was held constant at 2 mM. Values generated kinetic parameters in Table 1. **Table S6.** Velocities produced by PfkA with respective phosphate donors (ATP or GTP) for each trial without any effector, with the addition of NH_4_Cl(*) appearing to be required for maximal activity and subsequent combination NH_4_Cl(*) and other potential activating or inhibiting compound. These values were used to produce Fig. 4A–C. The effect of each effector was compared to the reaction with only NH_4_Cl(*), GTP/ATP and F6P which is considered 100% relative activity. Final concentration of each effector was 2 mM, F6P 2 mM and GTP/ATP 2 mM. **Table S7.** Inhibition assay for PfkA activity with varying concentrations of PP_i_ was done separately with another protein preparation. Note that PfkA once purified is not very stable and has the tendency to precipitate. The concentration of Mg^2+^ in the assay was reduced 50% to 2.5 mM to avoid precipitation with PP_i_. The reduction in Mg^2+^ along with potential precipitation of PfkA could explain the difference in velocities between Table S5 and Table S6. These values were used to produce Fig. 4D **Table S8.** Velocities produced by PfkB while varying the concentration of fructose to determine the *K*_m_ of the sugar while phosphate donor (ATP/GTP) concentration was held constant at 2 mM. Values generated kinetic parameters in Table 3. **Table S9.** Velocities produced by PfkB while varying the concentration of either ATP or GTP to determine the *K*_m_ of the phosphate donors while fructose concentration was held at 2 mM. Values generated kinetic parameters in Table 3. **Table S10.** Velocities produced by Aldoa while varying the concentration of FBP to determine the *K*_m_ of the sugar phosphate. **Table S11.** Velocities produced by Aldoa without any potential activating ions, with the addition of either a monovalent or divalent ion and a combination of both. The values were used to produce Fig. 11.

## Data Availability

All data generated or analyzed during this study are included in this published article, and additional files.

## Abbreviations

PFK: Phosphofructokinase
Aldoa: Fructose-bisphosphate aldolase
PP_i_: Pyrophosphate
Pfp: Pyrophosphate-dependent PFK
MOPS: (3-(*N*-Morpholino) propane-sulfonic acid
TFA: Trifluoroacetic acid
DHB: 2,5-Dihydroxybenzoic acid
XOD: Xanthine oxidase
PNPase: Purine nucleoside phosphorylase
MES: 2-Morpholinoethanesulfonic acid
HEPES: 2-[4-(2-Hydroxyethyl)piperazin-1-yl]ethane-1-sulfonic acid
