# Supplementary figures and images for: Kinetic characterization of annotated glycolytic enzymes present in cellulose-fermenting Clostridium thermocellum suggests different metabolic roles
Source: Biotechnol Biofuels Bioprod. 2023 Jul 12;16:112. doi: 10.1186/s13068-023-02362-8 (PMC10339645; doi:10.1186/s13068-023-02362-8)

# Additional File 1

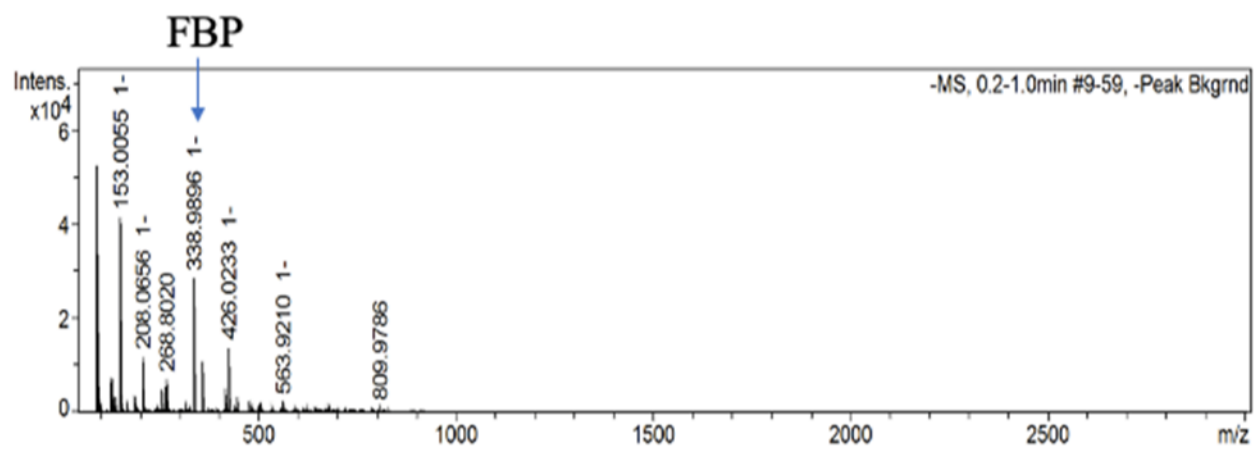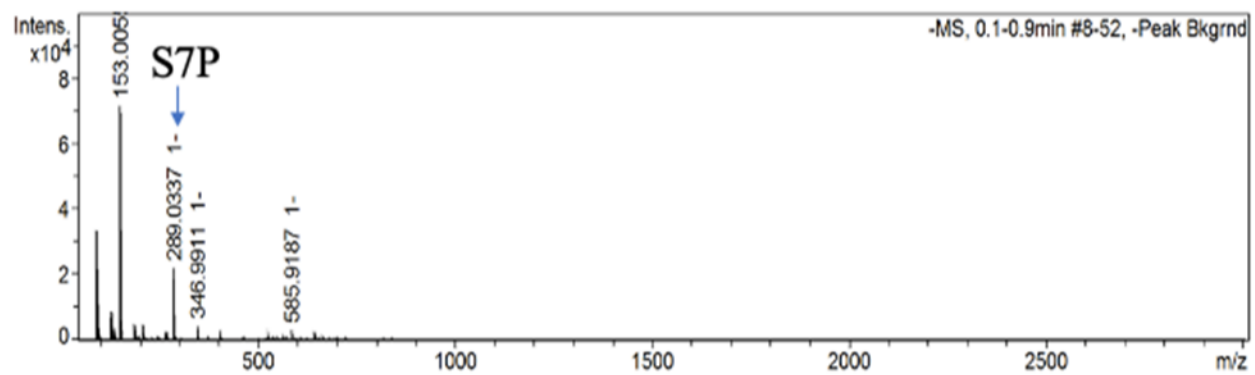

Supplement: Supplementary file 1 — Additional file 1. Mass spectrometry showing phosphorylated products observed with PfkA. Figure S1. The enzyme was able to phosphorylate fructose-6-phosphate(F6P), which has a theoretical molecular mass of 259.81 g/mol to Fructose-1,6-bisphosphate (MW = 340.116 g/mol), while utilizing GTP as the phosphate donor. The starting substrate(F6P) was low in relative abundance thus the peak at 259.0223 m/z was not seen on the chromatogram but was recorded in the spectrum deconvolution report. The peak for Fructose-1,6-bisphosphate (FBP) was seen at 338.989 m/z. Figure S2. The enzyme was not able to utilize sedoheptulose-7-phosphate (S7P, MW = 290.162 g/mol) as a substrate. The chromatogram only displays a peak for S7P, while there was no sign of sedoheptulose-1,7-bisphosphate (SBP,MW = 370.14 g/mol) on the chromatogram or spectrum deconvolution report. [file 13068_2023_2362_MOESM1_ESM.pdf]

Additional File 3

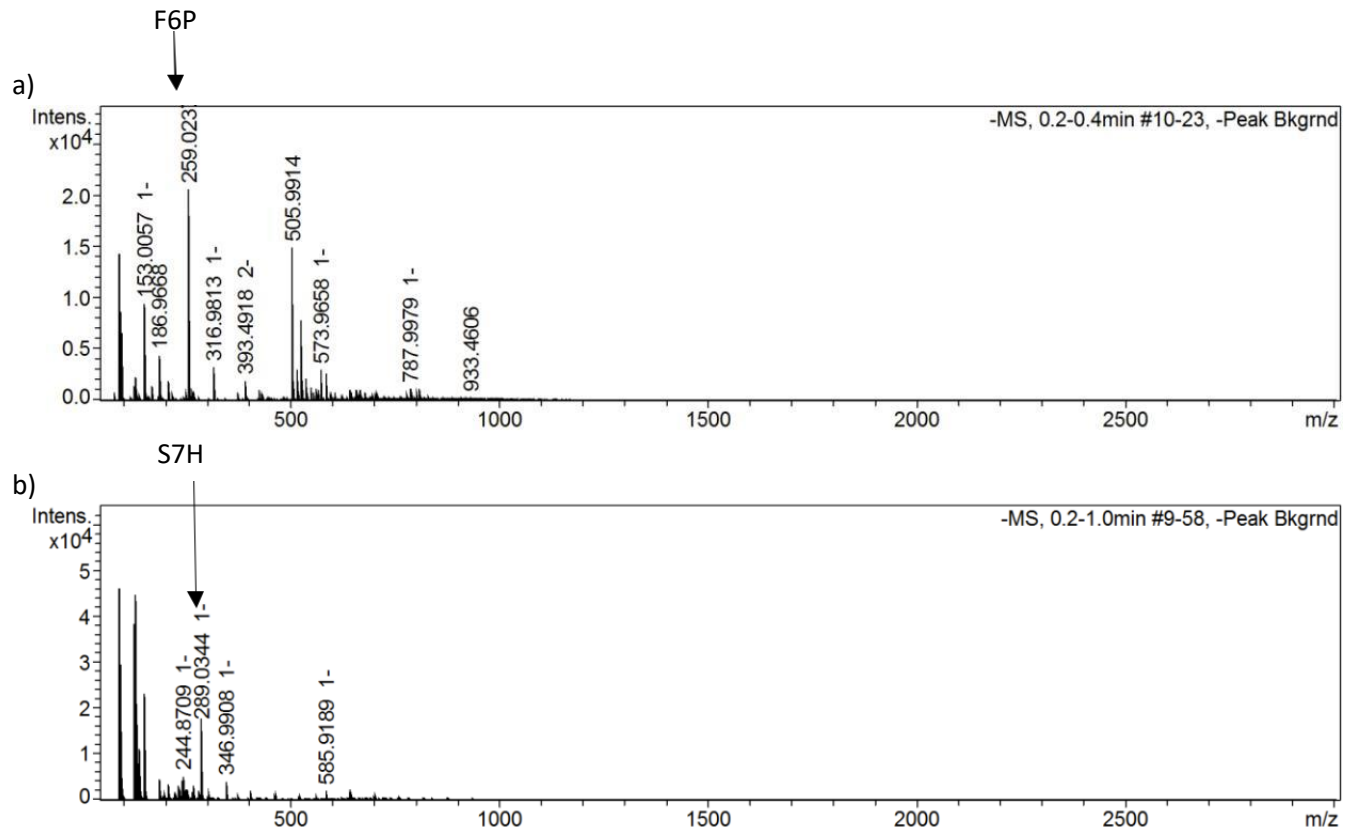

Supplement: Supplementary file 3 — Additional file 3. Mass spectrometry analysis of sugar phosphates present after the reaction with PfkB, being the phosphorylating enzyme in question. Figure S5. F6P, the starting substrate is the only sugar phosphate that can be identified on the chromatogram, thus FBP was not produced. Figure S6. S7H, the starting substrate is the only sugar phosphate that can be identified on the chromatogram, thus SBP was not produced. Both reactions were carried out with ATP as the phosphate donor. [file 13068_2023_2362_MOESM3_ESM.pdf]

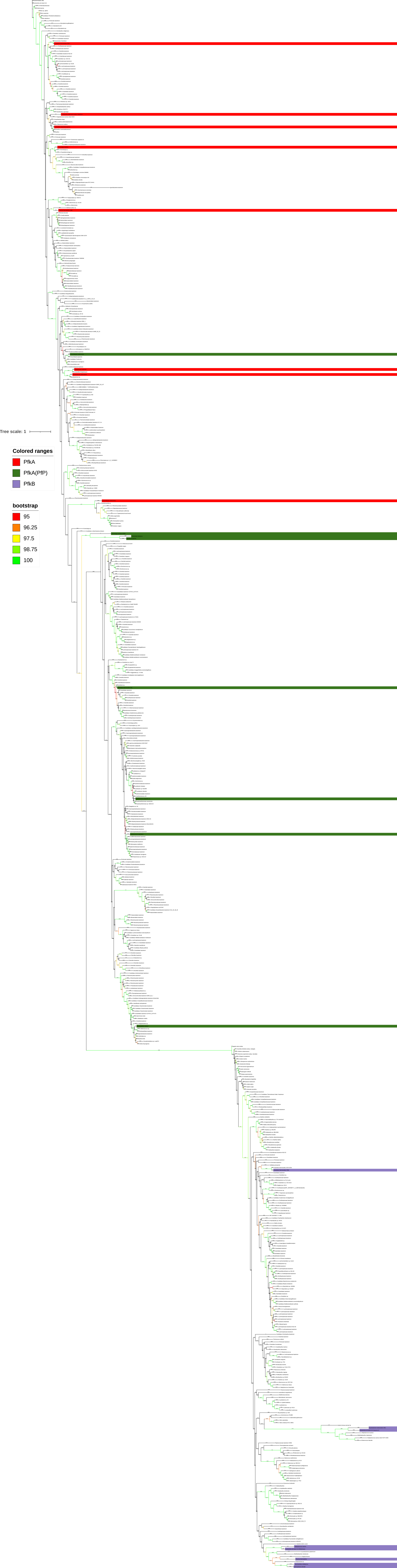

Supplement: Supplementary file 5 — Additional file 5. Phylogeny tree of PFK family. Figure S8. Phylogenetic analysis of 621 PFK sequences belonging Family A and B under the broader PFK superfamily by the maximum likelihood method. Characterized PfkA from different organisms were highlighted in red, Pfp highlighted in green and PfkB highlighted in purple. The tree is unrooted but Intestinirhabdus alba was the outgroup taxon drawn at the root. The tree is drawn to scale, with branch lengths measured in the number of substitutions per site. The probability of the branch being true was assessed by ultrafast bootstrap approximation, with values ≥ 95% considered reliable. [file 13068_2023_2362_MOESM5_ESM.pdf]
