# Supplementary material for: Kinetic characterization of annotated glycolytic enzymes present in cellulose-fermenting Clostridium thermocellum suggests different metabolic roles
Source: Biotechnol Biofuels Bioprod. 2023 Jul 12;16:112. doi: 10.1186/s13068-023-02362-8 (PMC10339645; doi:10.1186/s13068-023-02362-8)
Supplement: Supplementary file 2 — Additional file 2. FMichaelis–Menten kinetics of Pfp generated using SigmaPlot® 11.2 with data used to generate kinetic parameters and Pfp competitive assay. Figure S3. Activity of Pfp versus varied F6P concentration predicts a relatively low Km of 0.075 mM for F6P, but high Vmax, while PPi(2 mM) is used as a phosphate donor. Table S1. Data used to generate kinetics parameters for Pfp utilizing F6P + PPi in this study, Table 2. Figure S4. S7P is the substrate under investigation which is observed to have high Km of 3.236 and a much lower Vmax compared to assays with F6P. Table S2. Data used to generate kinetics parameters for Pfp utilizing S7P + PPi in this study, Table 2. Table S3. The rate of FBP production by Pfp at 45 °C was determined with F6P alone and in the presence of equimolar amounts of both F6P & S7P. There was approximately a 75% decrease in the rate of FBP production when both substrates were used in comparison to when F6P was the sole substrate, indicating that S7P could be competing for the active site. The velocity for the phosphorylation of solely S7P (2.12 mM) is also included in the figure for comparison to F6P (2 mM) phosphorylation. The production of Sedoheptulose-1,7-bisphosphate was confirmed by Mass Spectrometry [file 13068_2023_2362_MOESM2_ESM.pdf]

Additional File 2

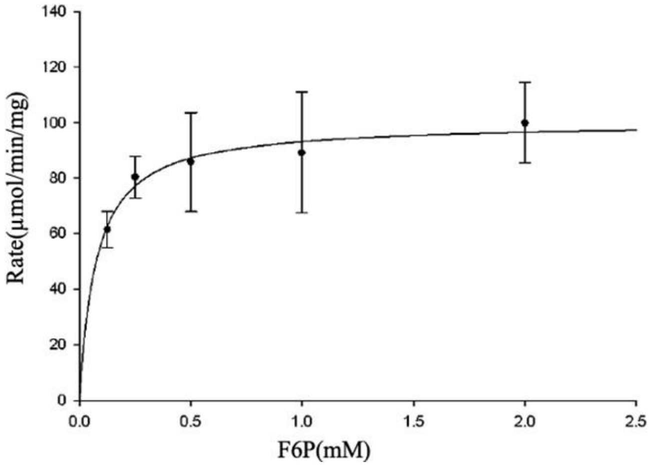

F6P (mM)	Velocity(μmol/min/mg)				Average (μmol/min/mg)
	Tr 1	Tr2	Tr3	Tr4	
2	122.203	127.700	74.863	74.844	99.902
1	129.347	124.146	54.724	48.522	89.185
0.5	122.663	110.140	54.604	55.574	85.745
0.25	92.061	94.355	67.699	67.284	80.350
0.125	75.936	68.7964	48.835	52.278	61.461

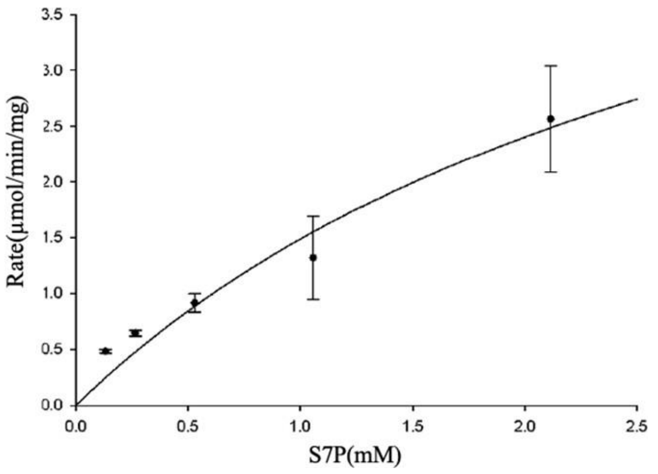

S7P (mM)	Velocity(µmol/min/mg)			Average (µmol/min/mg)
	Tr 1	Tr2	Tr3	
2.115	2.791	3.255	1.647	2.564
1.057	1.986	1.287	0.686	1.320
0.528	0.896	0.781	1.063	0.913
0.264	0.693	0.640	0.600	0.644
0.132	0.453	0.506	0.484	0.481

Substrate	Velocity(µmol/min/mg)			Average (µmol/min/mg)
	Tr 1	Tr2	Tr3	
F6P	31.197	25.235	20.956	25.796
S7P	2.791	3.255	1.647	2.564
F6P & S7P	4.293	8.338		6.316
