# Supplementary material for: Kinetic characterization of annotated glycolytic enzymes present in cellulose-fermenting Clostridium thermocellum suggests different metabolic roles
Source: Biotechnol Biofuels Bioprod. 2023 Jul 12;16:112. doi: 10.1186/s13068-023-02362-8 (PMC10339645; doi:10.1186/s13068-023-02362-8)
Supplement: Supplementary file 4 — Additional file 4. Amino acid alignments of class II FBP aldolases. Figure S7. Including the characterized FBP aldolase of C. thermocellum currently under investigation; as well as the other annotated FBP aldolase encoded by C. thermocellum (C. thermocellum_hyp). Thermus aquaticus, Thermotoga maritima, Giardia lamblia, Helicobacter pylori, Geobacillus stearothermophilus all belong to class IIB, while E. coli, Corynebacterium glutamicum, Saccharomyces cerevisiae, Euglena gracilis belong to class IIA. The conserved residues implicated in binding the Zn2+ (D) and monovalent cation (M) have been identified based off stucturalr studies done the aldolase of E. coli [52]. Segments highlighted in black, identical residues, while letters that are bold represent similar residues. The sequences were aligned with MAFFT and the figure prepared with ESPript. The percent similarity between C. thermocellum characterized Aldoa and each of the other aldolases were placed at the end of the alignment. [file 13068_2023_2362_MOESM4_ESM.pdf]

Additional File 4

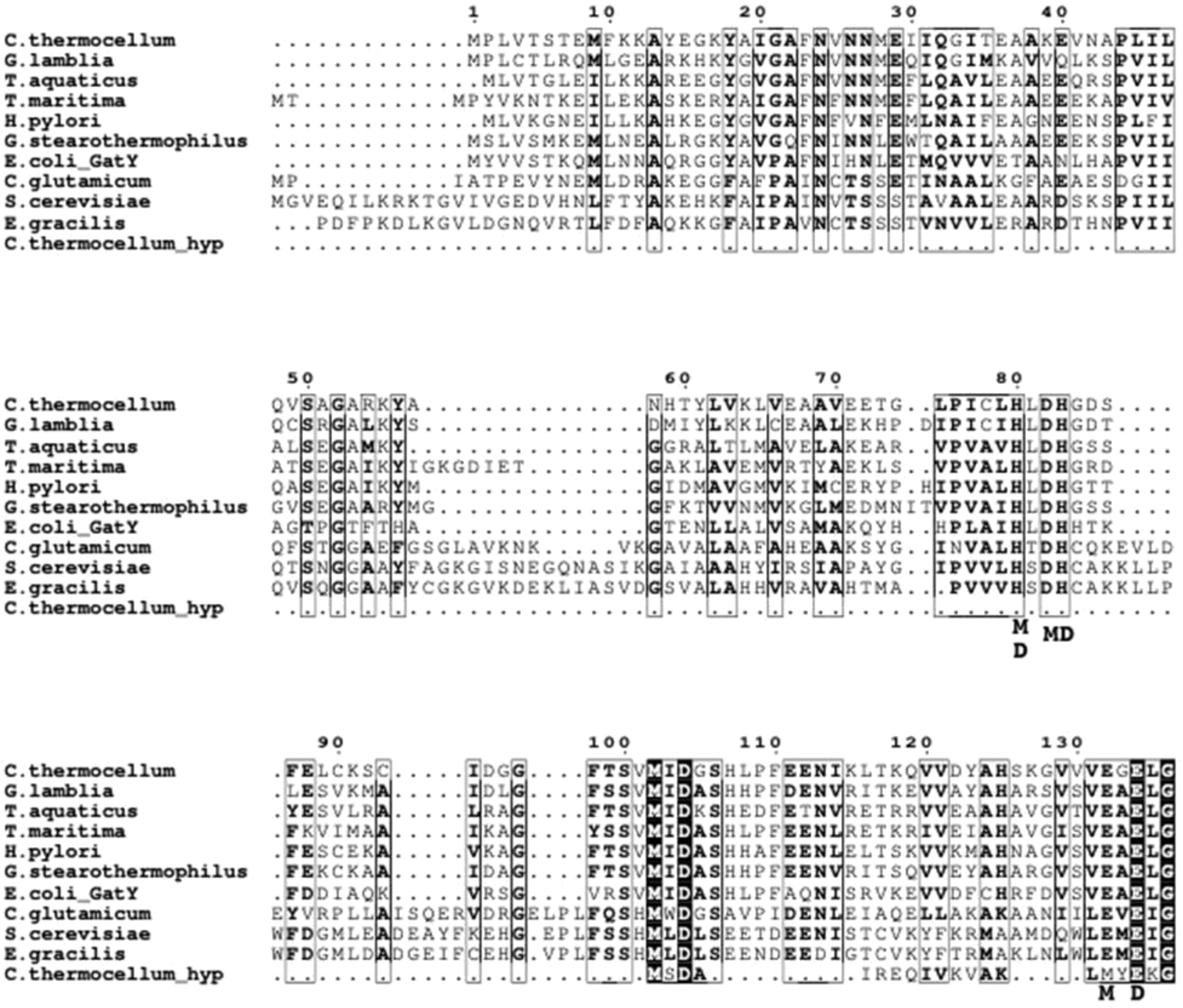

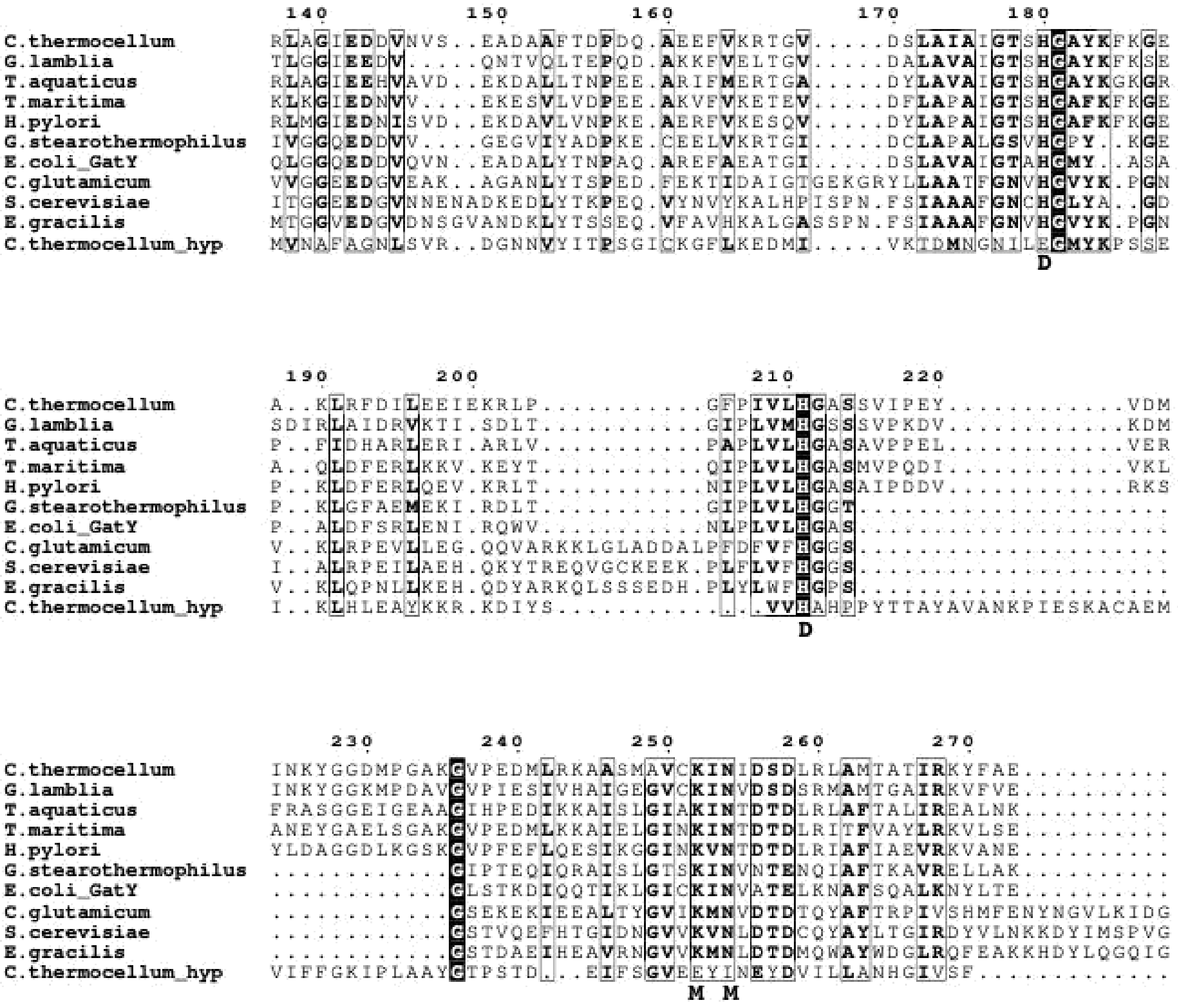
140 150 160 170 180
C.thermocellum RLAGIEDDVNVS..EADAAFTDPDQ.AEEFVKRTGV.....DSLAIAIGTSHGAYKFKGE
G.lamblia TLGGIEEDV.....QNTVQLTEPQD.AKKFVELTGV.....DALAVAIGTSHGAYKFKSE
T.aquaticus RLAGIEEHVAVD..EKDALLTNPEE.ARIFMERTGA.....DYLAVAIGTSHGAYKGKGR
T.maritima KLKGIEDNVV....EKESVLVDPEE.AKVFVKETEV.....DFLAPAIGTSHGAFKFKGE
H.pylori RLMGIEDNISVD..EKDAVLVNPKE.AERFVKESQV.....DYLAPAIGTSHGAFKFKGE
G.stearothermophilus IVGGQEDDVV....GEGVIYADPKE.CEELVKRTGI.....DCLAPALGSVHGPY..KGE
E.coli_GatY QLGGQEDDVQVN..EADALYTNPAQ.AREFAEATGI.....DSLAVAIGTAHGMY..ASA
C.glutamicum VVGGEEDGVEAK..AGANLYTSPED.FEKTIDAIGTGEKGRYLLAATFGNVHGVYK.PGN
S.cerevisiae ITGGEEDGVNNENADKEDLYTKPEQ.VYNVYKALHPISPN.FSIAAAFGNCHGLYA..GD
E.gracilis MTGGVEDGVDNSGVANDKLYTSSEQ.VFAVHKALGASSPN.FSIAAAFGNVHGVYK.PGN
C.thermocellum_hyp MVNAFAGNLSVR..DGNNVYITPSGICKGFLKEDMI.....VKTDMNGNILEGMYKPSSE
D
190 200 210 220
C.thermocellum A..KLRFDILEEIEKRLP...........GFPIVLHGASSVIPEY............VDM
G.lamblia SDIRLAIDRVKTI.SDLT...........GIPLVMHGSSSVPKDV............KDM
T.aquaticus P..FIDHARLERI.ARLV...........PAPLVLHGASAVPPEL............VER
T.maritima A..QLDFERLKKV.KEYT...........QIPLVLHGASMVPQDI............VKL
H.pylori P..KLDFERLQEV.KRLT...........NIPLVLHGASAIPDDV............RKS
G.stearothermophilus P..KLGFAEMEKI.RDLT...........GIPLVLHGGT..................
E.coli_GatY P..ALDFSRLENI.RQWV...........NLPLVLHGAS..................
C.glutamicum V..KLRPEVLLEG.QQVARKKLGLADDALPFDFVFHGGS..................
S.cerevisiae I..ALRPEILAEH.QKYTREQVGCKEEK.PLFLVFHGGS..................
E.gracilis V..KLQPNLLKEH.QDYARKQLSSSEDH.PLYLWFHGPS..................
C.thermocellum_hyp I..KLHLEAYKKR.KDIYS...............VVHAHPPYTTAYAVANKPIESKACAEM
D
230 240 250 260 270
C.thermocellum INKYGGDMPGAKGVPEDMLRKAASMAVCKINIDSDLRLAMTATIRKYFAE..........
G.lamblia INKYGGKMPDAVGVPIESIVHAIGEGVCKINVDSDSRMAMTGAIRKVFVE..........
T.aquaticus FRASGGEIGEAAGIHPEDIKKAISLGIAKINTDTDLRLAFTALIREALNK..........
T.maritima ANEYGAELSGAKGVPEDMLKKAIELGINKINTDTDLRITFVAYLRKVLSE..........
H.pylori YLDAGGDLKGSKGVPFEFLQESIKGGINKVNTDTDLRIAFIAEVRKVANE..........
G.stearothermophilus ............GIPTEQIQRAISLGTSKINVNTENQIAFTKAVRELLAK..........
E.coli_GatY ............GLSTKDIQQTIKLGICKINVATELKNAFSQALKNYLTE..........
C.glutamicum ............GSEKEKIEEALTYGVIKMNVDTDTQYAFTRPIVSHMFENYNGVLKIDG
S.cerevisiae ............GSTVQEFHTGIDNGVVKVNLDTDCQYAYLTGIRDYVLNKKDYIMSPVG
E.gracilis ............GSTDAEIHEAVRNGVVKMNLDTDMQWAYWDGLRQFEAKKHDYLQGQIG
C.thermocellum_hyp VIFFGKIPLAAYGTPSTD...EIFSGVEEYINEYDVILLANHGIVSF............
M M

```
                                    280       290       300
C.thermocellum           ........NPSHFDPRQYLGPARNAIKELVKHKIVNVLGCDGKA................
G.lamblia                ........HPEKFDPRDYLGPGRDAITEMLIPKI.KAFGSAGHAGDYKVVSLEEAKA...
T.aquaticus              ........NPKEFDPRKYLGPAREAVKEVVKSRM.ELFGSVGRA................
T.maritima               ........DKSQIDPRKIFKPVFEQVKEIVKERI.RIFGSSGKA................
H.pylori                 ........DKSQFDLRKFFSPAQLALKNVVKERM.KLLGSANKI................
G.stearothermophilus     ........DPNVYDPRKIIGPGRDAIKATVIGKM.REFGSSGKAAQ..............
E.coli_GatY              ........HPEATDPRDYLQSAKSAMRDVVSKVI.ADCGCEGRA................
C.glutamicum             .....EVGNKKAYDPRSYMKKAEQSMSERIIESC.QDLKSVGKTTSK.............
S.cerevisiae             NPEGPEKPNKKFFDPRVWVREGEKTMGAKITKSL.ETFRTTN..................
E.gracilis               NPEGPDKPNKNYYDPRKWIREAELGMLARVKVAF.KAVELPGGLKEFIGIP.........
C.thermocellum_hyp       ........GRDVFDAYFKLEAAEDIAKTLILSRL...LGGEKDLPENKLKELDDMRKKRR


C.thermocellum           ........ 100%
G.lamblia                .....WYK55.30%
T.aquaticus              ........51.62%
T.maritima               ........48.90%
H.pylori                 ........47.56%
G.stearothermophilus     ........44.87%
E.coli_GatY              ........38.51%
C.glutamicum             ........24.29%
S.cerevisiae             ........26.10%
E.gracilis               ........23.84%
C.thermocellum_hyp       TEVYW... N/A
```
