# Supplementary material for: Kinetic characterization of annotated glycolytic enzymes present in cellulose-fermenting Clostridium thermocellum suggests different metabolic roles
Source: Biotechnol Biofuels Bioprod. 2023 Jul 12;16:112. doi: 10.1186/s13068-023-02362-8 (PMC10339645; doi:10.1186/s13068-023-02362-8)
Supplement: Supplementary file 6 — Additional file 6: PfkA, PfkB and Aldoa kinetic data used to generate kinetic parameters in this study. Table S4. Velocities produced by PfkA while varying the concentration of either ATP or GTP to determine the Km of the phosphate donors while F6P was held at a concentration of 2 mM. Values generated kinetic parameters in Table 1. Table S5. Velocities produced by PfkA while varying the concentration of F6P to determine the Km of the sugar phosphate while phosphate donor (ATP/GTP) concentration was held constant at 2 mM. Values generated kinetic parameters in Table 1. Table S6. Velocities produced by PfkA with respective phosphate donors (ATP or GTP) for each trial without any effector, with the addition of NH4Cl(*) appearing to be required for maximal activity and subsequent combination NH4Cl(*) and other potential activating or inhibiting compound. These values were used to produce Fig. 4A–C. The effect of each effector was compared to the reaction with only NH4Cl(*), GTP/ATP and F6P which is considered 100% relative activity. Final concentration of each effector was 2 mM, F6P 2 mM and GTP/ATP 2 mM. Table S7. Inhibition assay for PfkA activity with varying concentrations of PPi was done separately with another protein preparation. Note that PfkA once purified is not very stable and has the tendency to precipitate. The concentration of Mg2+ in the assay was reduced 50% to 2.5 mM to avoid precipitation with PPi. The reduction in Mg2+ along with potential precipitation of PfkA could explain the difference in velocities between Table S5 and Table S6. These values were used to produce Fig. 4D Table S8. Velocities produced by PfkB while varying the concentration of fructose to determine the Km of the sugar while phosphate donor (ATP/GTP) concentration was held constant at 2 mM. Values generated kinetic parameters in Table 3. Table S9. Velocities produced by PfkB while varying the concentration of either ATP or GTP to determine the Km of the phosphate donors while fructo [file 13068_2023_2362_MOESM6_ESM.pdf]

Additional File 6

Phosphate donor (mM)	Velocity(µmol/min/mg) ATP			Average U/mg	Velocity(µmol/min/mg) GTP			Average U/mg
	Tr 1	Tr 2	Tr 3		Tr1	Tr2	Tr3	
2	188.739	217.206	181.289	195.745	261.287	266.569	263.307	263.721
1	115.290	100.767	104.641	106.899	212.031	211.872	223.291	215.731
0.5	63.123	55.093	87.600	68.605	171.110	179.630	190.340	180.360
0.25	67.467	55.706	52.275	58.482	165.823	147.102	152.532	155.152
0.125	28.018	25.287	33.869	29.058	99.002	97.547	99.108	98.552

F6P (mM)	Velocity(µmol/min/mg) GTP			Average U/mg	Velocity(µmol/min/mg) ATP			Average U/mg
	Tr 1	Tr 2	Tr 3		Tr1	Tr2	Tr3	
8	442.365	441.772	460.057	448.064				
6	727.205	715.271	394.090	612.188				
4	626.336	609.613	512.361	582.770				
2	248.381	220.467	262.146	243.664	147.352	128.743	137.573	137.889
1	115.881	109.596	119.895	115.124	109.511	96.603	84.843	96.985
0.5	50.465	43.054	46.430	46.650	66.687	70.080	85.999	74.255
0.25	25.689	22.021	20.335	22.681	48.388	50.566	57.916	52.290
0.2	13.229	15.732	16.419	15.126				
0.125	16.106	13.527	14.390	14.674	41.307	43.225	38.841	41.124

	Velocity(µmol/min/mg) ATP			Average U/mg	Relative activity (%)	Velocity(µmol/min/mg) GTP			Average U/mg	Relative activity (%)
Effector	Tr 1	Tr 2	Tr 3			Tr 1	Tr 2	Tr 3		
No effector	4.76	16.91	15.71	12.48		43.26	60.78	49.83	51.29	
NH_4Cl(*)	187.44	215.75	179.87	164.12	100	259.61	264.95	261.59	220.92	100
	142.50	123.40	135.78			152.29	166.70	220.39		
Malic Acid*	172.40	178.23	65.19	138.61	84	236.00	253.00	238.89	242.63	110
Citric acid*	166.10	139.04	162.83	155.99	95	285.24	246.43	262.57	264.75	120
G6P*	187.30	155.12	70.35	137.59	84	223.48	231.33	173.52	209.44	95
KCl*	145.23	105.96	137.86	129.68	79	254.58	238.91	214.60	236.03	107
AMP*	195.72	144.20	106.43	148.78	91	220.82	247.38	233.89	234.03	106
ADP*	170.21	175.45	209.85	185.17	113	217.33	260.87	246.00	241.40	109
PEP*	102.93	91.30	37.49	77.24	47	96.78	103.67	93.91	98.12	44%

PP_i added (mM)	Velocity(µmol/min/mg) ATP			Average U/mg	Relative activity (%)	Velocity(µmol/min/mg) GTP			Average U/mg	Relative activity (%)
	Tr 1	Tr 2	Tr 3			Tr 1	Tr 2	Tr 3		
0	32.99	32.13	30.41	31.85	100	16.73	16.82	15.84	16.47	100
0.05	6.69	7.32	6.28	6.76	21.24	2.81	4.62	5.05	4.16	25.27
0.1	2.86	3.73	2.35	2.98	9.35	0.86	0.37	0.16	0.46	2.82
0.2	1.27	1.58	1.30	1.39	4.36	0.73	0.48	1.07	0.76	4.62
0.4	1.41	0.75	1.52	1.22	3.846	0.6	0.59	0.30	0.50	3.02

Fructose (mM)	Velocity(µmol/min/mg) ATP			Average U/mg	Velocity(µmol/min/mg) GTP			Average U/mg
	Tr 1	Tr 2	Tr 3		Tr1	Tr2	Tr3	
2	179.547	147.401	122.021	149.656	176.051	151.278	143.600	156.976
1	87.428	90.240	89.260	88.976	129.125	128.232	167.519	141.625
0.5	51.603	79.704	54.011	61.772	84.961	90.004	72.498	82.487
0.25	39.717	40.401	44.887	41.668	51.501	52.582	62.491	55.525
0.125	21.920	22.521	18.403	20.948	39.045	36.521	39.646	38.404

Phosphate donor (mM)	Velocity(µmol/min/mg) ATP			Average U/mg	Velocity(µmol/min/mg) GTP			Average U/mg
	Tr 1	Tr 2	Tr 3		Tr1	Tr2	Tr3	
2	74.873	107.837	100.640	94.450	146.966	137.910	56.199	113.692
1	92.886	92.791	79.024	88.234	94.104	97.875	104.655	98.878
0.5	53.139	52.769	34.340	46.749	82.566	83.533	107.361	91.153
0.25	59.969	52.473	54.502	55.648	79.204	83.385	81.852	81.480
0.125	34.973	32.576	25.801	31.117	39.181	35.641	39.158	37.993

FBP (mM)	Velocity(µmol/min/mg)			Average (µmol/min/mg)
	Tr 1	Tr 2	Tr 3	
2	26.928	24.469	26.311	25.903
1	21.211	24.644	26.566	24.140
0.5	19.401	22.092	21.457	20.983
0.25	17.766	18.573	19.660	18.666
0.125	12.209	12.092	12.127	12.143

	Velocity(µmol/min/mg)			Average U/mg	Relative activity (%)
Effector	Tr 1	Tr 2	Tr 3		
No effector	16.047	15.370	11.417	14.278	100
$MgCl_2$	14.600	13.687	11.672	13.320	93
$ZnCl_2$	33.030	35.230	32.912	33.724	236
NH_4Cl + $MgCl_2$	26.599	24.507	26.101	25.736	180
$ZnCl_2$ + NH_4Cl	21.038	18.276	22.924	20.746	145
$ZnCl_2$ + KCl	17.826	21.505	22.886	20.739	145
$MgCl_2$ + KCl	10.821	10.323	10.948	10.697	75
